# The first endemic West African vertebrate family – a new anuran family highlighting the uniqueness of the Upper Guinean biodiversity hotspot

**DOI:** 10.1186/1742-9994-11-8

**Published:** 2014-02-03

**Authors:** Michael F Barej, Andreas Schmitz, Rainer Günther, Simon P Loader, Kristin Mahlow, Mark-Oliver Rödel

**Affiliations:** 1 Museum für Naturkunde, Leibniz Institute for Research on Evolution and Biodiversity, Invalidenstrasse 43, D-10115 Berlin, Germany; 2 Department of Herpetology and Ichthyology, Natural History Museum of Geneva, CP 6434, 1211 Geneva 6, Switzerland; 3 Department of Environmental Sciences (Biogeography), University of Basel, Klingelbergstr. 27, Basel 4056, Switzerland

**Keywords:** Amphibia, Anura, Ranoidae, Natatanura, Odontobatrachidae fam. nov., Petropedetidae, Biodiversity hotspot, Higher level systematics, Molecular phylogeny, Osteology, West Africa

## Abstract

**Background:**

Higher-level systematics in amphibians is relatively stable. However, recent phylogenetic studies of African torrent-frogs have uncovered high divergence in these phenotypically and ecologically similar frogs, in particular between West African torrent-frogs versus Central (*Petropedetes*) and East African (*Arthroleptides* and *Ericabatrachus*) lineages. Because of the considerable molecular divergence, and external morphology of the single West African torrent-frog species a new genus was erected (*Odontobatrachus*). In this study we aim to clarify the systematic position of West African torrent-frogs (*Odontobatrachus*). We determine the relationships of torrent-frogs using a multi-locus, nuclear and mitochondrial, dataset and include genera of all African and Asian ranoid families. Using micro-tomographic scanning we examine osteology and external morphological features of West African torrent-frogs to compare them with other ranoids.

**Results:**

Our analyses reveal Petropedetidae (*Arthroleptides*, *Ericabatrachus*, *Petropedetes*) as the sister taxon of the Pyxicephalidae. The phylogenetic position of *Odontobatrachus* is clearly outside Petropedetidae, and not closely related to any other ranoid family. According to our time-tree estimation *Odontobatrachus* has been separated from other frog lineages since the Cretaceous (90.1 Ma; confidence interval: 84.2-97.1 Ma). Along with this molecular evidence, osteological and external diagnostic characters recognize West African torrent-frogs as distinct from other ranoids and provide strong support for the necessity of the recognition of a new family of frogs. This is the only endemic vertebrate family occurring in the Upper Guinea biodiversity hotspot.

**Conclusion:**

Based on molecular and morphological distinctiveness, the West African torrent-frog *Odontobatrachus natator* is allocated to a newly described anuran family*.* The discovery of an endemic vertebrate family in West Africa highlights the Upper Guinean forests as an outstanding, but highly endangered biodiversity hotspot.

## Background

The availability of large-scale phylogenies in recent years has focused attention on higher-level phylogenetic relationships in amphibians [1-7]. Frost et al. [1] introduced almost 30 higher level taxa (above family level) in anuran systematics and one additional caecilian family (Chikilidae) was recently described from India [8]. However, in the course of major phylogenetic studies two families described by Frost et al. [1] (Cryptobatrachidae, Thoropidae) have been subsequently recognized as synonyms [9,10]. For anurans, with the exception of the discovery of the enigmatic burrowing frog *Nasikabatrachus sahyadrensis* (Nasikabatrachidae) from India [11], the recognition of new families in the last two decades have referred mainly to rearrangements, reassessments of subfamilies or splits of speciose genera, which have revealed the appropriate taxonomic placement of taxa [1,9,12,13]. So despite some nomenclatural modifications and a single exceptional finding (see above), large-scale molecular data did not make significant modifications to family level classification of anurans.

In the course of a phylogenetic study of sub-Saharan torrent-frogs, previously considered to be integral part of a single genus (*Petropedetes* Reichenow, 1874), the West African species (*P. natator*) was shown to be highly divergent [14]. Barej et al. [14] recovered three distinct lineages, all having distinct geographic distributions. Based on molecular and morphological differences these authors consequently described the new genus *Odontobatrachus*, endemic to Guinea, Sierra Leone, Liberia and Ivory Coast. The genus description was based on a number of morphological synapomorphies and a deep molecular divergence of *Odontobatrachus* from Central African *Petropedetes* and East African *Arthroleptides*[14]. Surprisingly, *Odontobatrachus* was placed outside the family Petropedetidae, challenging the monophyly of this family. It also could not be assigned to any of the other groups sampled in this study [14]. We aim at resolving the higher-level phylogenetic relationships of the genus *Odontobatrachus* by including molecular samples of representatives of all African and Asian ranoids. Furthermore, using micro-tomographic scanning and staining techniques, we examine the osteological and external morphological features to compare *Odontobatrachus* with other ranoid families.

## Results and discussion

**Figure 1:**
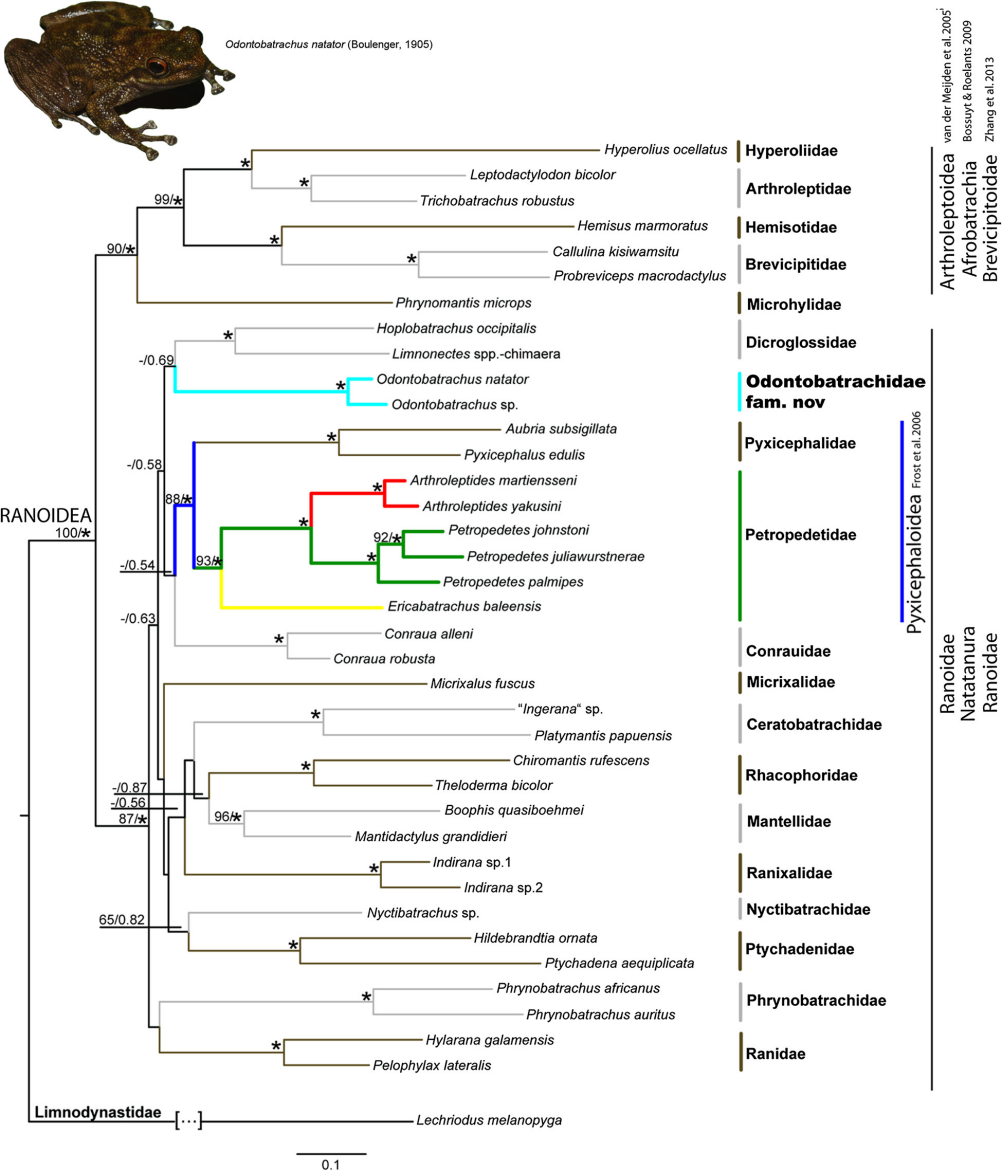
**Phylogeny of ranoid frogs.** Phylogeny of ranoid frogs based on mitochondrial and nuclear data. Numbers along branches indicate bootstrap values as obtained using RAxML 7.0.4 and Bayesian posterior probabilities. Asterisks point to maximum support under both methods (ML: 100/PP: 1.00). Colour codes reflect distinct lineages of African torrent-frogs Petropedetidae (green = *Petropedetes*, Central Africa; red = *Arthroleptides*, East Africa; yellow = *Ericabatrachus,* Ethiopia) and West African torrent-frogs Odontobatrachidae fam. nov. (turquoise = *Odontobatrachus*, West Africa).

Combined analyses [a total of 3474 bp] of 3 mitochondrial [*12S*, *16S*, *cytb*: 1472 bp] and 3 nuclear genes [*SIA*, *rag1*, *BDNF*: 2002 bp] resulted in a topology consistent with recent large-scale phylogenetic studies [1,3,7]. Both Maximum Likelihood (ML) and Bayesian Inference (BI) revealed two major clades in the superfamily Ranoidea (Figure 1). The first clade consisted of the sub-Saharan families Hyperoliidae, Arthroleptidae, Hemisotidae and Brevicipitidae (in former studies referred to Afrobatrachia *sensu* Frost et al. [1], Arthroleptoidea *sensu* van der Meijden et al. [15] or Brevicipitoidea *sensu* Zhang et al. [7]), and the globally distributed family Microhylidae. We follow the argumentation of [7] concerning the priority of Brevicipitoidea Bonaparte, 1850, over Arthroleptoidea [16] and Afrobatrachia [1]. This major clade is consistent with current phylogenies [1,3,7,17]. Brevicipitoidea + Microhyloidea form a sister group to all other families in the second major clade (epifamily Ranoidae *sensu* van der Meijden et al. [15]). Our phylogenetic analyses placed the genus *Odontobatrachus* with strong support in the epifamily Ranoidae within the superfamily Ranoidea. With the exception of the families Mantellidae and Petropedetidae, all included neobatrachian families containing more than one representative (on species or genus level) showed maximum support in BI and ML (Mantellidae: BS = 96, PP = 1.00; Petropedetidae: discussed below). While relationships in the Brevicipitoidea + Microhylidae were well resolved, basal nodes in the Ranoidae remained unresolved in both analyses, forming a large polytomy with a single exception (Petropedetidae + Pyxicephalidae). Only Zhang et al. [7] have shown in their mitogenomic study moderate support values for family level relationships within Ranoidae. African torrent-frogs of the genera *Arthroleptides* and *Petropedetes* formed a maximally supported clade (BS = 100, PP = 1.00) and together with the Ethiopian monotypic and endemic genus *Ericabatrachus* they represent the family Petropedetidae (BS = 93, PP = 1.00). Our analyses revealed strong support for Petropedetidae as the sister taxon to the family Pyxicephalidae (*Aubria*, *Pyxicephalus*), grouped in the Pyxicephaloidea *sensu* Frost et al. [1]. However, Frost et al. [1] also included *Conraua* and *Indirana* in their family Petropedetidae, both now being placed in distinct families [3]. The genus *Odontobatrachus* was positioned clearly outside the clade of Pyxicephalidae + Petropedetidae. A close relationship of *Odontobatrachus* with the pyxicephalid subfamily Cacosterninae was previously rejected by Barej et al. [14], and the inclusion of a second representative of the subfamily Pyxicephalinae supported this result. Additional analyses with constrained topologies placing *Odontobatrachus* sister to the families Pyxicephalidae and Petropedetidae (*Ericabatrachus*, *Arthroleptides*, *Petropedetes*) or within the family Petropedetidae (as the sister taxon to a clade consisting of *Arthroleptides* + *Petropedetes* or sister to any of the three genera respectively) were clearly rejected (Additional file 1: 1.3). *Odontobatrachus* could not be clearly assigned to any family within the Ranoidae. Bayesian analyses indicate a weakly supported sister relationship of *Odontobatrachus* to the family Dicroglossidae (PP = 0.69). ML analyses weakly resolved (BS < 30, not shown) *Odontobatrachus* as sister group to the Phrynobatrachidae. Dicroglossidae are known from a single species in sub-Saharan savannahs, otherwise being species rich on the Arabian Peninsula, the Indian subcontinent and Asia [18-21]. Phrynobatrachidae is found across sub-Saharan Africa [22]. *Odontobatrachus* is shown to be separated from all clades by a deep branch, forming a distinct and highly supported lineage (Figure 1).

Recent large-scale phylogenies [1,3,7] did not include *Odontobatrachus natator*. In these analyses, African torrent-frogs were represented by Central and East African taxa, and based on the assumption that *Odontobatrachus natator* is a petropedetid, the species has been placed in the family Petropedetidae. Scott [23] included *O. natator* in her simultaneous analyses of molecular and morphological data. However, only morphological data of *O. natator* was available to Scott [23], and this dataset did not place the West African taxon outside the genus *Petropedetes*. In contrast, our molecular results clearly deviate from this conclusion with West African torrent-frogs *Odontobatrachus natator* placed outside the family Petropedetidae. The distinctiveness of *Odontobatrachus natator* is also supported by various osteological characters (see Additional file 1: 2.2).

Our molecular time scale estimates the split of *Odontobatrachus* in the Cretaceous (90.1 Ma; confidence interval: 84.2-97.1 Ma; see Additional file 1: 1.2). The dates in our timescale are also temporally comparable with other major splits within the epifamily Ranoidae that are recognized at the family level rank in anuran classification [24,25]. Although, there is a lack of resolution in the basal nodes among African and Asian ranids our dating results supports the high distinctiveness of the West African torrent-frogs.

Geographical events that may support a historical scenario of long-term isolation of the West African area, as suggested by *Odontobatrachus*, are known from the literature. From the late Jurassic/Early Cretaceous onwards rifting of the proto-South Atlantic started and this was accompanied with development of rift systems in West and Central Africa [26]. At the same time, climate warming (peaking in the Upper Cretaceous) led to transgressions entering local basins [27] and as large parts of the continental shelf were flooded, only temporary land bridges connected the West African craton with Central Africa [28]. At the same time vegetation changed and closed canopy forests arose [29-32]. It can be therefore speculated that the West African torrent-frogs might have evolved in a geographically isolated area during ongoing vegetation changes, explaining the restricted distribution of this lineage. Other scenarios for example, extinction in other biogeographic regions of a formerly more widespread *Odontobatrachus,* with remaining extant lineages in West Africa, are possible but seem less likely.

In summary, despite weakly resolved basal relationships of *Odontobatrachus*, our results clearly support the distinctiveness of West African torrent-frogs in relation to all other families of the higher taxon Natatanura or Ranoidae (Figure 1). The problem of insufficient taxon sampling as a cause of long branches is known [33], however, our taxon sampling was relatively complete. The relative and absolute branch lengths of *Odontobatrachus* compared to its tentatively assigned nearest sister group correspond to those differences exhibited between other Ranoidae families in our analyses, and supports the distinct status of West African torrent-frogs *Odontobatrachus*.

Taxonomic changes, as proposed in our study, need to be carefully assessed in order to sustain stability of classifications. Vences et al. [34] proposed criteria for the recognition of (higher level) taxa (but see [35]). Most important criteria *sensu*[34] are: (i) monophyly, (ii) clade stability and (iii) phenotypic diagnosability. We demonstrate that all these aspects are applicable in the case of the West African torrent-frogs *Odontobatrachus*. As (i) the case concerns a single genus (with currently one described species and potentially additional taxa), (ii) recognized to be distinct by a phylogenetic approach using optimization methods with a dense taxon and genetic sampling, and (iii) morphological differentiation from all other families [see below]. A detailed differentiation between the morphologically most similar petropedetid genera is given in the Additional file 1: 2.2. Consequently, we place the West African torrent-frogs *Odontobatrachus* in an own, new family, described below.

Amphibia Linnaeus, 1758

Lissamphibia Haeckel, 1866

Anura Rafinesque, 1815

Neobatrachia Reig, 1958

Odontobatrachidae fam. nov.

Type genus: *Odontobatrachus* Barej, Rödel, Loader & Schmitz, 2014

Type species: *Petropedetes natator* Boulenger, 1905

### Diagnosis

The new family is distinguished from all other families within the Ranoidea on the basis of molecular characters (see Figure 1) and the following combination of morphological characters (see also Additional file 1: 2).

### Osteological characters

#### ***Skull***

**Figure 2:**
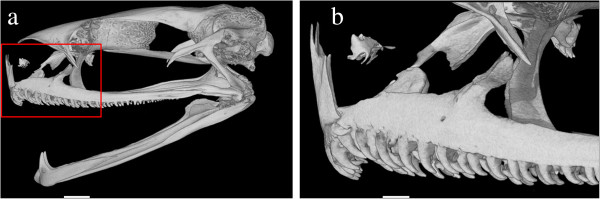
**CT-scan of *Odontobatrachus *skull.** Computer tomographic scan in lateral view of *Odontobatrachus natator* skull (ZMB 78203) **a**: lateral view of left side, lower jaw virtually rotated to open the mouth, red square defines close-up shown in b; scale bar = 1.5 mm; **b**: close up of the anterior part of the maxilla; scale bar = 0.5 mm. The images highlighting the tusk-like odontoids on the lower mandible as well as curved and pointed pre- and maxillary teeth; skull virtually isolated from the remaining skeleton.

Nasals large, rectangular, in median contact; nasals overlapping sphenethmoid; ventral sphenethmoid with considerable forward extension; anterior ramus of pterygoid not reaching neopalatines and planum orbitale; posterior process of vomer connected to main mass of vomer, vomerine teeth present; premaxillary and maxillary teeth distinct, curved backwards and pointed (Figure 2); tusk-like odontoid on lower mandible; zygomatic ramus longer than otic ramus.

#### ***Pectoral girdle***

Base of omosternum convex; medial edges of coracoids not overlapping; posterior edge of sternum wide and weakly serrated; metasternum short and hourglass shaped, with broad bony stylus; clavicle thickness approximately equal along entire length.

#### ***Axial skeleton***

Centrum of presacral vertebra VIII rather amphicoelous; metacarpal of digit II in breeding males not enlarged and spike-like; shape of terminal phalanges T- to slightly Y-shaped on hand and on foot.

#### ***Hyolaryngeal apparatus***

Hyoid plate wider than long; hyale without a free flange towards jaw, hyale with a small and hooked anterior process; hyoglossal sinus deeper than anterior border of base of anterolateral processes; anterolateral processes T-shaped with a broad base, the posterolateral processes long, reaching up to the middle of the posteromedial processes; distance between anterior edges of posteromedial processes less than one time of their width; small cartilaginous bridge between the enlarged anterior ends of the posteromedial processes; calcifications or ossifications only present in posteromedial processes.

#### ***External morphology***

Tympanum indistinct, smaller than eye diameter; skin granular with enlarged ridges (roundish to elongate); extensive webbing; males with pair of external vocal sacs; positioned ventrolaterally; nuptial excrescences velvety in breeding males; femoral glands present in males only.

#### ***Tadpole morphology***

Flattened body shape, sucker-like mouthparts with enlarged labials.

### Differential diagnosis

A combination of the above mentioned characters distinguishes the new family from remaining taxa within the superfamily Ranoidea. However, a few characters are sufficient to exclude most of them (based on [23,36-42]: Osteology: maxillaries with teeth (absent in Brevicipitidae, Microhylidae); vomerine teeth present (absent in Micrixalidae); presence of mandibular tusks (present otherwise only in Ceratobatrachidae, Dicroglossidae, Nyctibatrachidae, Phrynobatrachidae, Pyxicephalidae, Ranixalidae); zygomatic ramus longer than otic ramus (shorter in Mantellidae, Petropedetidae); crista parotica cartilaginous (mineralized in Conrauidae, Dicroglossidae, Pyxicephalidae); terminal phalanges Y- to T-shaped (blunt or pointed in Hemisotidae, Dicroglossidae, curved in Ptychadenidae); external morphology: medium to large sized frogs reaching 65 mm snout-vent length (< 30 mm in Micrixalidae); dorsal skin granular with short dorsal and dorsolateral glandular ridges (skin smooth with dorsolateral glandular ridge in Micrixalidae); tympanum indistinct (distinct in Ranixalidae); presence of femoral glands (shared with Mantellidae, Nyctibatrachidae, Petropedetidae, Phrynobatrachidae, Pyxicephalidae, Ranixalidae); absence of gular gland (present in Hyperoliidae); absence of lateral line system (present in Conrauidae, Nyctibatrachidae); hyoid with long posterolateral process (short or absent in Arthroleptidae, Hyperoliidae, Mantellidae); presence of nuptial pads (absent in Nyctibatrachidae).

The morphologically most similar family Petropedetidae can be differentiated from Odontobatrachidae fam. nov. by the following morphological characters: presence of tusk-like odontoids on mandible (absent in Petropedetidae), presence of lateral vocal sacs in males (absent or median in Petropedetidae). A detailed differential diagnosis to Central and East African torrent frogs (*Petropedetes* and *Arthroleptides*) including an osteological differentiation is provided in Additional file 1: 2.

### Phylogenetic definition

The new family comprises all anurans which are more closely related to *Odontobatrachus natator* than to members of other ranoid families. Current content: one genus, *Odontobatrachus*.

### Distribution

The single included genus is known from the Upper Guinean forests in Guinea [43-47], Sierra Leone [48,49], Liberia [50], and western Ivory Coast [51], where frogs usually occur close to streams with strong currents and cascades or rapids.

### Diversity

At present a single species is described, *Odontobatrachus natator* (Boulenger, 1905). However, Barej et al. [14] already recognized a higher diversity in this lineage and a more detailed taxonomic analysis of the family throughout the distribution range in the Upper Guinea forests is in preparation (M.F. Barej et al. unpubl. data).

### Etymology

The name refers to the Greek words όδoύς (odous = tooth, genitive: odóntos) and βατραχoσ (batrachos = frog) and points to the exceptionally long maxillary teeth and large tusks on lower jaws in these frogs (Figure 2).

Remark: In accordance with article 8.5 of the International code of Zoological Nomenclature (International Commission on Zoological Nomenclature [52]) the present publication (LSID: urn:lsid:zoobank.org:pub:DFB71831-37B0-4292-8193-74C8045CD35B) and nomenclatural act (LSID: urn:lsid:zoobank.org:act:464214AA-FB13-4626-B04E-55DB0DE94B2A) have been registered in ZooBank.

### Hotspot West Africa

‘West African Forests’ have been recognized as one of the major biodiversity hotspots on global scale [53,54]. This hotspot comprises two parts, the “Upper” and “Lower Guinea” forests. Only recently the West African amphibian fauna was confirmed to be distinct from those of the Central African parts of the Guineo-Congolian forest block [55]. Despite a high number of endemic amphibians (especially forest species), the West African biodiversity hotspot [55] comprises only four endemic anuran genera (*Morerella*, *Nimbaphrynoides*, *Odontobatrachus*, *Pseudhymenochirus*) [14,39,56,57], the lowest number of endemic genera compared to the other biogeographic realms of the African continent (Central Africa: 17; eastern Africa 13, southern Africa 11; Additional file 1: 3). West African endemics are known from various different taxonomic groups, although diversity in many of them is still not completely understood (e.g. bats [58,59], primates [60]; plants [61-63], fresh water fish [64], birds, amphibians and mammals [65,66]). However, among terrestrial vertebrates in western Africa, there are no endemic bird genera, only a single endemic skink genus (*Cophoscincopus*) and six endemic mammal genera, including the pygmy hippopotamus and the red colobus (*Hexaprotodon*, *Procolobus*, *Liberiictis, Micropotamogale*, *Leimacomys*, *Dephomys*[67-69]). So far no endemic vertebrate taxon, higher than genus level, is known for the Upper Guinea forests of West Africa.

West African torrent-frogs have for a long time been assumed to form a distinct sub-Saharan lineage (Amiet in Perret [70]), although they cover similar ecological niches as Central and East African torrent-frogs [71-74]. More than a century after the species description [48], West African torrent-frogs are now assigned to a distinct family, Odontobatrachidae fam. nov., based on molecular and morphological characters. The placement of these frogs outside the Petropedetidae [14] or any other ranoid family, as shown here, provides important evidence that a deep diverging endemic lineage occurs in West Africa. Although Western Africa has the lowest number of anuran genera (4 endemics and 27 in total), all biogeographical areas in Africa show similar numbers of families (East Africa = 15, West, Central, South Africa = 14; data extracted from [75]; Additional file 1: 3). Interestingly however, only South Africa and West Africa have an endemic anuran family (namely Heleophrynidae in South Africa [76]).

Both endemic families share similarities in being relatively species poor relative to their age compared to other anuran families [25,76-80]. Whether the distinction of endemic families and their diversity in western and southern Africa reflects something about the specific biogeographical history of these areas, or our incomplete understanding of African amphibians in general, is difficult to assess. Despite this uncertainty, the description of a family with a highly restricted distribution is of biogeographical and conservation significance. Conservation of genetic diversity [81-83], as well as species richness is increasingly seen as an important consideration [84-88]. Therefore, the conservation of West African forests for amphibians is of high priority and highlights more broadly the biological importance of this area. This is particularly necessary given conservation of Upper Guinean forest habitats is generally poor, and forest cover is rapidly shrinking [89], with negative consequences for many different taxonomic groups [90-94]; but see [95] in an area rich in endemic species [53].

Despite a burst of taxonomic activity in the description of Upper Guinean amphibians, with more than a dozen described taxa since the year 2000 [1] and many additional species awaiting description (Rödel et al. unpubl data), the finding of a distinct evolutionary anuran lineage reflects how incomplete our knowledge within an apparently well-studied part of this continent really is. The extraordinary finding of a new vertebrate family (Amphibia, Anura, Odontobatrachidae fam. nov.), endemic to western Africa highlights the peculiarity of the Upper Guinea hotspot.

## Conclusion

West African torrent-frogs have been recently recognized as a distinct genus [14]. This study demonstrates the distinctiveness of this lineage among all currently known families in the Ranoidea clade. Preliminary dating points to an origin of the lineage in the Cretaceous, a period of high diversification of family lineages in Ranoidea [25]. Molecular results distinguish the lineage comprising West African torrent-frogs from all known Ranoidea families. A comparison of osteological characters of this lineage with torrent-frogs from Central and East Africa, family Petropedetidae, further supports its distinctiveness. Consequently, a new family, Odontobatrachidae fam. nov., is described for West African torrent-frogs. The genus *Odontobatrachus* is monotypic but undescribed species have been identified and their description is pending [14]. Present findings of an endemic frog family in West Africa reflect the importance of this biodiversity hotspot, as South Africa is the only other African region with an endemic anuran family.

## Methods

### Taxon sampling and phylogenetics

Presented data comprise representatives of all families currently grouped in the superfamily Ranoidea [3,7,15,96]. More specifically, our focus was on the taxon Natatanura [25,97,98] or Ranoidae [7,15]. Included taxa are members of the families (respective geographic origin: Africa = AF, Asia = AS, Madagascar = MG): Arthroleptidae (AF), Brevicipitidae (AF), Ceratobatrachidae (AS), Conrauidae (AF), Dicroglossidae (AF/AS), Hemisotidae (AF), Hyperoliidae (AF), Mantellidae (MG), Micrixalidae (AS), Microhylidae (AF), Nyctibatrachidae (AS), Petropedetidae (AF), Phrynobatrachidae (AF), Ptychadenidae (AF)*,* Pyxicephalidae (AF), Ranidae (AF/AS), Ranixalidae (AS), Rhacophoridae (AF/AS), *Odontobatrachus* (*incertae sedis sensu* Barej et al. [14]; AF). The monotypic genus *Ericabatrachus,* not available to Barej et al. [14], has been recently demonstrated to be closely related to the African torrent-frog family Petropedetidae [99], and has been added here in order to cover the whole phylogenetic diversity of this group. A list including all voucher identifications and GenBank [100] numbers is provided in the Additional file 1: 1.1. A member of the family Limnodynastidae has been included as the single outgroup taxon in the analysis. The family Limnodynastidae, present in Australia and New Guinea, is also part of the Neobatrachia and therein is assigned to the Myobatrachoidea (*sensu* Bossuyt & Roelants [25]).

### Molecular analyses

Applied methods followed [14] with regard to lab protocols and data analyses. The final data matrix consisted of three nuclear [Seven-in-Absentia (*SIA*: 396 bp), Recombination Activation gene 1 (*rag1*: 930 bp) and Brain-derived neurotrophic factor gene (*BDNF*: 676 bp)] and three mitochondrial genes [(*12S* rRNA: 346 bp, *16S* rRNA: 538 bp, cytochrome b gene *cytb*: 588 bp)]. Bayesian Inference (MrBayes, version 3.21 [101]) and Maximum Likelihood (RAxML version 7.0.4 [102] using the rapid hill climbing algorithm following Stamatakis et al. [103]) were applied to access phylogenetic relationships. Bootstrap analyses (BS) with 1000 pseudoreplicates in the ML analysis were used to evaluate the relative branch support in the phylogenetic analysis. Bayesian analyses were run for 5 million generations using four chains sampling every 100 generations, with a burn-in of 1000 trees. Clades with posterior probabilities (PP) ≥ 95% were considered strongly supported. Stationarity has been checked with Tracer V1.5 [104]. Seven alternative tree topologies were evaluated against our optimal tree topology using the approximately unbiased test (AU) [105] and the multiple comparisons test (SH, Shimodaira–Hasegawa test) [106] as implemented in Treefinder Version of March 2011 [107].

### Osteological analyses

A i) morphological and anatomical diagnosis of the West African lineage and ii) an in-depth anatomic comparison to the externally similarly looking *Petropedetes* and *Arthroleptides* was conducted. In order to achieve this a non-destructive micro-tomographic analysis based on four West African vouchers (*Odontobatrachus natator* males: ZMB 78203, ZMB 78222, ZMB 78243; female: ZMB 78216) and representatives of Central and East African torrent-frogs (see Additional file 1: 2.1) was carried out. Images were generated using a Phoenix nanotom X-ray|s tube at 70–80 kV and 90–100 μA for total body scans, 90–100 kV and 90–150 μA for close-ups of the skull respectively, generating 1000 projections per scan. Effective voxel size ranged between 17–23 μm for total body scans and 8–16 μm for close-ups of the skull. The cone beam reconstruction was performed using the datos|x-reconstruction software (GE Sensing & Inspection Technologies GMBH phoenix|x-ray) and the data were visualized in VG Studio Max 2.1. To back up the validity of CT-scan interpretations, we additionally cleared and double stained ZMB 78222, according to a modified method of Dingerkus & Uhler [108]. A complete description of osteological characters, a figure of the double stained hyolaryngeal apparatus and plates of osteological characters are provided in Additional file 1: 2.1 and 2.2.

### Dating estimates

Dating was performed with the software package BEAUti and BEAST 1.7.5 [109] under the Yule Process speciation model [110,111] and the relaxed clock model [112]. Mean heights and 95% credibility interval values for node time estimates were generated from a total of 3474 bp of coding and non-coding genes (Additional file 1: 1.2). An ultrametric tree was generated with Mesquite Version 2.75 [113] as the starting tree for Beast analyses. The following outgroup taxa were added to our dating approach, to ensure known calibration constraints on internal nodes: *Latimeria*, *Hynobius*, *Andrias* and *Calyptocephalella* (GenBank numbers see Additional file 1: 1.2).

Calibration points are based on fossil records and published review data (splits 5 – 10 are based on published data by Bossuyt & Roelants [25]): 1. split coelacanth – tetrapoda (420 Ma [114]), 2. split Anura – Caudata (230 Ma; based on fossils of *Triadobatrachus*[115]), 3. split hynobiid and cryptobranchid salamanders (161 Ma; based on cryptobranchid fossil [116]), 4. split *Calyptocephalella* and *Lechriodus* (min. 53 Ma; based on *Calyptocephalella* fossil [117]), 5. split Ranoidea – remaining Neobatrachia (161 Ma), 6. split Ranoidea – Myobatrachoidea (Limnodynastidae; 119 Ma), 7. split Microhyloidae + Brevicipitoidea – Ranoidae (117 Ma), 8. split Brevicipitoidea (*sensu* Zhang et al. [7]) – Microhyloidae (102 Ma), 9. split Arthroleptidae – Hyperoliidae (87.7 Ma), 10. split Pyxicephalidae – Petropedetidae (81.1 Ma). Calibrations have been applied solely to a reduced dataset as basal relationships within the Ranoidae remain poorly or even not resolved. Included taxa refer to five outgroup taxa (*Latimeria*, *Hynobius*, *Andrias, Calyptocephalella, Lechriodus*), Microhylidae, Brevicipitoidea (Arthroleptidae, Brevicipitidae, Hemisotidae, Hyperoliidae) and Ranoidae (Dicroglossidae, Petropedetidae, Pyxicephalidae and the new family).

## Competing interests

The authors declare that they have no competing interests.

## Authors’ contributions

MFB, AS, MOR designed the study. Molecular data collection was performed by MFB, AS and SPL. Analyses were carried out by MFB and AS. KM generated CT-scans and prepared respective figures. Osteological analyses were performed by RG and MFB. Data interpretation and preparation of the manuscript was done by MFB, AS, RG, MOR and SPL. The final manuscript was written by MFB. All authors read, commented on and approved the final manuscript.

## Supplementary Material

Additional file 1 **The first endemic West African vertebrate family – a new anuran family highlighting the uniqueness of the Upper Guinean biodiversity hotspot [1. Molecular analyses (GenBank numbers, molecular dating, topology test); 2. Osteological analyses (*Odontobatrachus* osteology, Petropedetidae vs. Odontobatrachidae fam. nov.); 3. Amphibian diversity in African realms 4. References of Additional file].** Click here for file
