# Supplementary material for: The first endemic West African vertebrate family – a new anuran family highlighting the uniqueness of the Upper Guinean biodiversity hotspot
Source: Front Zool. 2014 Feb 3;11:8. doi: 10.1186/1742-9994-11-8 (PMC3925359; doi:10.1186/1742-9994-11-8)
Supplement: Additional file 1 — The first endemic West African vertebrate family – a new anuran family highlighting the uniqueness of the Upper Guinean biodiversity hotspot [1. Molecular analyses (GenBank numbers, molecular dating, topology test); 2. Osteological analyses ( Odontobatrachus osteology, Petropedetidae vs. Odontobatrachidae fam. nov.); 3. Amphibian diversity in African realms 4. References of Additional file]. [file 1742-9994-11-8-S1.pdf]

# Additional file: The first endemic West African vertebrate family – a new anuran family highlighting the uniqueness of the Upper Guinean biodiversity hotspot

Michael F. Barej[a,*], Andreas Schmitz[b], Rainer Günther[a], Simon P. Loader[c], Kristin Mahlow[a], Mark-Oliver Rödel[a]

[a]Museum für Naturkunde, Leibniz Institute for Research on Evolution and Biodiversity, Invalidenstrasse 43, D-10115 Berlin, Germany

[b]Natural History Museum of Geneva, Department of Herpetology and Ichthyology, C.P. 6434, 1211 Geneva 6, Switzerland

[c]University of Basel, Department of Environmental Sciences (Biogeography), Klingelbergstr. 27, Basel 4056, Switzerland

[*]Corresponding author: michael@barej.de

## Content: page:

**1. Molecular analyses**

**1.1 GenBank accession numbers** 2

**1.2 Molecular dating** 4

**1.3 Topology test** 5

**2. Osteological analyses**

**2.1 *Odontobatrachus* osteology** 6

**2.2 Osteological characters distinguishing Petropedetidae and Odontobatrachidae fam. nov.** 10

**3. Amphibian diversity in African realms** 25

**4. References** 26

# 1. Molecular analyses

### 1.1 GenBank accession numbers

Previous studies of African and Asian ranids lacked representatives across all families and genera. This study aimed to provide as comprehensive a sampling as possible to resolve the appropriate taxonomic rank of *Odontobatrachus*. Initial analyses tentatively indicated the grouping of *Odontobatrachus* and the family Dicroglossidae (*Hoplobatrachus;* occurring in Africa and Asia). A second member of Dicroglossidae family, belonging to *Limnonectes*, was generated as a chimaera (*Limnonectes poilani* and *L. laticeps*) to form a clade of the family Dicroglossidae, to more thoroughly test the grouping of *Odontobatrachus*. Where possible, two distinct genera or distantly related species of one genus within one family have been included. Widely distributed anuran families with genera of Asian and African origin have been included in our analyses. Museum abbreviations of voucher and tissue samples included are as follows: The Natural History Museum, London, United Kingdom (BMNH); California Academy of Sciences, San Francisco, USA (CAS); Natural History Museum of Geneva, Switzerland (MHNG); Staatliches Museum für Naturkunde, Stuttgart, Germany (SMNS); Vietnam National Museum of Nature, Hanoi, Vietnam (VNMN); Zoologisches Forschungsmuseum Alexander Koenig, Bonn, Germany (ZFMK); Museum für Naturkunde, Berlin, Germany (ZMB); Zoological Natural History Museum, Addis Ababa University, Ethiopia (ZNHM-AAU). Remaining material (Be, JR) refers to field collections. Remarks: (1) Sequences of *Limnonectes poilani* ($x^1$) were not assigned individual collection numbers; respective publications are provided [1,2] and listed in the references. (2) A BLAST search in GenBank [3] of specimen CAS 246787 revealed highest similarity with "*Ingerana*" *baluensis* (Ceratobatrachidae) and is consequently maintained as "*Ingerana*" sp. All GenBank numbers are provided in Table A1.

**Table A1 List of taxa included in this study.** List of taxa included in this study including voucher ID and locality data (country codes: CI = Cote d'Ivoire, CM = Cameroon, BJ = Benin, ET = Ethiopia, GA = Gabon, GN = Guinea, ID = Indonesia, IN = India, KH = Cambodia, LR = Liberia, MG = Madagascar, MM = Myanmar, TZ = Tanzania, VN = Viet Nam). Sequences marked in italic letters were extracted from GenBank and refer to deviating vouchers. **New accession numbers added upon acceptance of the manuscript.**

| family | *genus* | *species* | country | coll # | *12S* | *16S* | *cytb* | *BDNF* | *SIA* | *rag1* |
|---|---|---|---|---|---|---|---|---|---|---|
| Arthroleptidae | *Leptodactylodon* | *bicolor* | CM | ZMB 78460 | KF991244 | KF991264 | KF991306 | KF991283 | KF991343 | KF991325 |
| Arthroleptidae | *Trichobatrachus* | *robustus* | CM | ZMB 77508 | KF991245 | KF991265 | KF991307 | KF991284 | KF991344 | KF991326 |
| Brevicipitidae | *Callulina* | *kisiwamsitu* | TZ | BMNH 2002.45 | AY531840 | AY531863 | FJ998380 | KF991285 | KF991345 | KF990043 |
| Brevicipitidae | *Probreviceps* | *macrodactylus* | TZ | BMNH 2002.754 | FN81100 | FN811049 | FN811094 | KF991286 | KF991346 | KC632525 |
| Ceratobatrachidae | *"Ingerana"* | sp. | MM | CAS 246787 | KF991246 | KF991266 | KF991308 | KF991287 | KF991347 | KF991327 |
| Ceratobatrachidae | *Platymantis* | *papuensis* | ID | ZMB 70131 | KF991247 | KF991267 | KF991309 | KF991288 | KF991348 | KF991328 |
| Conrauidae | *Conraua* | *alleni* | GN | ZMB 78433 | KF693285 | KF693389 | KF693669 | KF693487 | KF693549 | KF693609 |
| Conrauidae | *Conraua* | *robusta* | CM | ZMB 78427 | KF693281 | KF693385 | KF693665 | KF693483 | KF693545 | KF693605 |
| Dicroglossidae | *Hoplobatrachus* | *occipitalis* | GN | ZMB 79256 | KF991248 | KF991268 | KF991310 | KF991289 | KF991349 | KF991329 |
| Dicroglossidae | *Limnonectes* | *laticeps/poilani* | --- | --- | *DQ283378* | *DQ283378* | *AB488856* | *AB489065* | *DQ282841* | *AB488960* |
| Hemisotidae | *Hemisus* | *marmoratus* | TZ | BMNH 2005.1385 | AY531831 | AY531854 | FN811075 | *AB612014* | KF991350 | *AB612013* |
| Hyperoliidae | *Hyperolius* | *ocellatus* | CM | MHNG 2715.58 | KF693275 | KF693379 | KF693659 | KF693477 | KF693539 | KF693599 |
| Limnodynastidae | *Lechriodus* | *melanopyga* | ID | ZMB 79235 | KF991249 | KF991269 | KF991311 | KF991290 | KF991351 | KF991330 |
| Mantellidae | *Boophis* | *quasiboehmei* | MG | ZMB 79257 | KF991250 | KF991270 | KF991312 | KF991291 | KF991352 | KF991331 |
| Mantellidae | *Mantidactylus* | *grandidieri* | MG | JR153 | KF991251 | KF991271 | *DQ235432* | KF991292 | KF991353 | KF991332 |
| Micrixalidae | *Micrixalus* | *fuscus* | IN | no Voucher | KF991252 | KF991272 | KF991313 | KF991293 | --- | KF991333 |
| Microhylidae | *Phrynomantis* | *microps* | CI | SMNS 9773 | AY531855 | AY531832 | FN563039 | *AB611980* | KF991354 | *AB611977* |
| Nyctibatrachidae | *Nyctibatrachus* | sp. | IN | no Voucher | KF991253 | KF991273 | KF991314 | KF991294 | KF991355 | KF991334 |
| Odontobatrachidae | *Odontobatrachus* | *natator* | GN | ZMB 78211 | KF693286 | KF693390 | KF693670 | KF693488 | KF693550 | KF693610 |
| Odontobatrachidae | *Odontobatrachus* | sp. | GN | ZMB 78317 | KF693290 | KF693394 | KF693674 | KF693492 | KF693554 | KF693614 |
| Petropedetidae | *Arthroleptides* | *martiensseni* | TZ | ZFMK 77306 | KF693292 | KF693396 | KF693676 | KF693494 | KF693556 | KF693616 |
| Petropedetidae | *Arthroleptides* | *yakusini* | TZ | BMNH 2005.567 | JX546941 | JX546965 | KF693680 | KF693502 | KF693563 | KF693622 |
| Petropedetidae | *Ericabatachus* | *baleensis* | ET | ZNHM-AAU-A2013-001 | KF938362 | KF938365 | KF991315 | KF991295 | KF991356 | KF938370 |
| Petropedetidae | *Petropedetes* | *johnstoni* | CM | ZFMK 87710 | KF693314 | GU256028 | KF693690 | KF693515 | KF693576 | KF693635 |
| Petropedetidae | *Petropedetes* | *juliawurstnerae* | CM | MHNG 2713.19 | KF693323 | KF693419 | KF693693 | KF693517 | KF693578 | KF693637 |
| Petropedetidae | *Petropedetes* | *palmipes* | GA | NCSM 76813 | KF693329 | KF693424 | KF693695 | KF693519 | KF693580 | KF693639 |
| Phrynobatrachidae | *Phrynobatrachus* | *africanus* | CM | MHNG 2715.45 | KF693278 | KF693382 | KF693662 | KF693480 | KF693542 | KF693602 |
| Phrynobatrachidae | *Phrynobatrachus* | *auritus* | CM | MHNG 2715.89 | KF693277 | KF693381 | KF693661 | KF693479 | KF693541 | KF693601 |
| Ptychadenidae | *Hildebrandtia* | *ornata* | BJ | ZMB 79258 | KF991254 | KF991274 | KF991316 | KF991296 | KF991357 | KF991335 |
| Ptychadenidae | *Ptychadena* | *aequiplicata* | LI | ZMB 79259 | KF991255 | KF991275 | KF991317 | KF991297 | KF991358 | KF991336 |
| Pyxicephalidae | *Aubria* | *subsigillata* | LI | ZMB 79260 | KF991256 | KF991276 | --- | KF991298 | KF991359 | KF991337 |
| Pyxicephalidae | *Pyxicephalus* | cf. *edulis* | BJ | Be119 | KF991257 | KF991277 | KF991318 | KF991299 | KF991360 | KF991338 |
| Ranidae | *Hylarana* | *galamensis* | GN | ZMB 79262 | KF991258 | KF991278 | KF991319 | KF991300 | KF991361 | KF991339 |
| Ranidae | *Pelophylax* | *lateralis* | KH | ZFMK 92555 | KF991259 | KF991279 | KF991320 | KF991301 | KF991362 | *EF088273* |
| Ranixalidae | *Indirana* | sp.1 | IN | no Voucher | KF991260 | KF991280 | KF991321 | KF991302 | KF991363 | KF991340 |
| Ranixalidae | *Indirana* | sp.2 | IN | no Voucher | KF991261 | KF991281 | KF991322 | KF991303 | KF991364 | KF991341 |
| Rhacophoridae | *Chiromantis* | *rufescens* | CM | ZFMK87811 | KF991262 | KF991282 | KF991323 | KF991304 | KF991365 | *GQ204605* |
| Rhacophoridae | *Theloderma* | *bicolor* | VN | VNMN 1394 | KF991263 | JX046475 | KF991324 | KF991305 | KF991366 | KF991342 |

## 1.2 Molecular dating

Calibrations points used were based on data extracted from GenBank sequences, respective GenBank numbers are provided in Table A2. Tentative divergence time estimates provided an age estimate of the West African family Odontobatrachidae fam. nov. of 90.10 Ma [95% confidence interval (CI Ma) 84.19-97.06 Ma, Figure A1]. The split between the families Pyxicephalidae and Petropedetidae has been calculated as 81.25 Ma (CI Ma: 79.32-83.15 Ma). The origin of the family Odontobatrachidae dates back to major splits within the higher-level taxon Ranoidae [4]. Interestingly, molecular timescales could provide a means to estimate taxonomic rank for amphibians [5]. Avise and Liu' [5] estimation for amphibians fits well within the taxonomic rank of family for Odontobatrachidae.

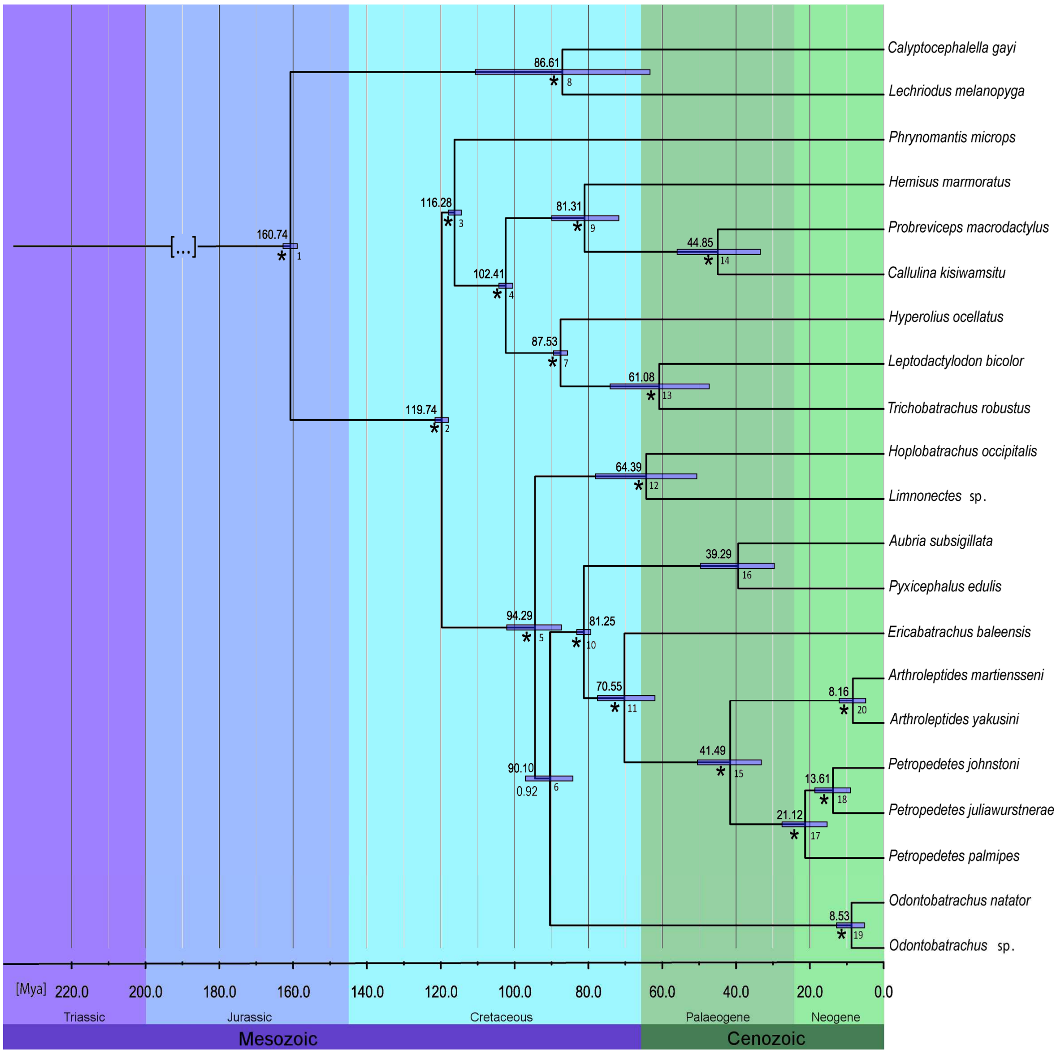


**Figure A1 Timetree of Ranoidea.** Timetree of the Ranoidea based on mitochondrial and nuclear sequences. Given are mean estimate values (above the 95% congruence interval bar, CI), posterior probabilities (below CI) and node numbers behind the respective node. Divergence times are provided in Table A2. The tree is rooted with hierarchical outgroups Sarcopterygii (*Latimeria*), Caudata (Cryptobranchidae, Hynobiidae); outgroups not shown (for GenBank numbers of outgroups see Table A3).

**Table A2 Divergence time estimate of Ranoidea.** Divergence time estimate, 95% confidence/credibility interval in million years (CI MA). Node numbers refer to Figure A1.

| Node | CI MA |
|---|---|
| 1 | 158.84-162.73 |
| 2 | 117.97-121.55 |
| 3 | 114.48-118.00 |
| 4 | 100.49-104.28 |
| 5 | 87.32-102.10 |
| 6 | 84.19-97.06 |
| 7 | 85.64-89.46 |
| 8 | 63.34-110.63 |
| 9 | 71.74-89.94 |
| 10 | 79.32-83.15 |
| 11 | 62.00-77.52 |
| 12 | 50.63-78.15 |
| 13 | 47.29-74.13 |
| 14 | 33.41-55.96 |
| 15 | 33.14-50.42 |
| 16 | 29.60-49.65 |
| 17 | 15.31-27.55 |
| 18 | 8.99-18.66 |
| 19 | 5.18-12.75 |
| 20 | 4.89-12.08 |

**Table A3 List of outgroup taxa included in the timetree.** List of GenBank sequences applied for molecular dating estimates.

| | family | genus | species | mitochondrial genes | BDNF | SIA | rag1 |
|---|---|---|---|---|---|---|---|
| Osteichthyes: Sarcopterygii | Latimeriidae | *Latimeria* | *menadoensis* | **AP006858** | **JQ073170** | **---** | **JQ073290** |
| Caudata | Cryptobranchidae | *Andrias* | *japonicus / davidianus* | **NC004926** | **EU275889** | **---** | **AY583346** |
| Caudata | Hynobiidae | *Hynobius* | *yiwuensis* | **NC020649** | **HM037750** | **---** | **HM037725** |
| Anura | Limnodnastidae | *Calyptocephalella* | *gayi* | **JF703228** | **JF703236** | **DQ282893** | **EF107334** |

## 1.3 Topology tests

Likelihood values of seven alternative topologies were tested against the unconstraint optimal tree (Fig. 1). Results of the AU-test and the SH-test are provided in Table A4. The genus *Odontobatrachus* was placed either basal to the Petropedetidae (Table A4.1), sister to any genus within the Petropedetidae (Table A4.2-A4.4) or sister to a clade comprising Central and East African torrent-frog genera (*Petropedetes* + *Arthroleptides*; Table A4.5). All placements related to the family Petropedetidae were clearly rejected. However, due to a weak resolution in basal nodes (lacking resolved relationships within the Natatanura), topologies placing Odontobatrachidae fam. nov. sister to other families cannot be excluded completely – as shown in the potential grouping with Conrauidae, which cannot be statistically significantly rejected.

However, alternative topologies show lower probability values and were not shown to have any strong support in any phylogenetic analyses (compare Table A4.1, A4.6 and A4.7).

**Table A4 Log-likelihoods and P values of AU test and SH test of the unconstrained tree and seven alternative topologies, with focus on the family Petropedetidae.**

| # | Alternative topologies | -ln *L* | AU-Test | SH-Test |
|---|---|---|---|---|
| | unconstrained topology | 38414.13 | 0.97 | 0.99 |
| 1 | Odontobatrachidae fam. nov. sister to Petropedetidae (*Ericabatrachus* (*Petropedetes, Arthroleptides*)) | 38428.86 | <0.05 | <0.05 |
| 2 | Odontobatrachidae fam. nov. sister to *Ericabatrachus* | 38842.84 | <0.001 | <0.01 |
| 3 | Odontobatrachidae fam. nov. sister to *Arthroleptides* | 38576.46 | <0.001 | <0.001 |
| 4 | Odontobatrachidae fam. nov. sister to *Petropedetes* | 38568.83 | <0.001 | <0.001 |
| 5 | Odontobatrachidae fam. nov. sister to Petropedetidae s.str. (*Petropedetes*, *Arthroleptides*) | 38441.99 | <0.001 | <0.001 |
| 6 | Odontobatrachidae fam. nov. sister to Pyxicephalidae (*Aubria, Pyxicephalus*) | 38427.52 | <0.05 | <0.05 |
| 7 | Odontobatrachidae fam. nov. sister to Conrauidae (*Conraua*) | 38419.45 | 0.15 | 0.14 |

## 2. Osteological analyses

### 2.1 *Odontobatrachus* osteology

We provide a detailed analysis of osteological characters based on CT scans (mainly ZMB 78203; supplemented with ZMB 78216, ZMB 78222, ZMB 78243) and double stained (ZMB 78222) material of *Odontobatrachus natator*.

Skull: Paired frontoparietals rectangular, their posterior ends fused and affiliated to the otoccipital (*sensu* Trueb [6]), frontoparietals width 51% of length, anterior margins fragmented, without contact to the nasals (Figure A2a); sphenethmoid in dorsal view visible between the anterior margin of the frontoparietals and the posterior margin of the nasals; paired nasals large and roofing entire forepart of the skull (Figure A2a), posterolateral process strongly expressed and with a dorsal groove; nasals not reaching the maxillary at any point; occipital condyles roundish, well developed and distant from one another; otic crest and epiotic eminence clearly pronounced; pars media plectra long and rod-shaped, the pars interna plectra clubbed; pterygoid triradiate, its anterior ramus long, not reaching the neopalatine, its medial ramus rather short and robust with its roundish end attached to the braincase, pterygoid not in contact with the alary process of the parasphenoid, posterior ramus long, narrow and attached to the quadrate (Figure A2c, A13a); squamosal strongly built, zygomatic ramus long and tapered, otic ramus shorter, wider and not firmly connected to the crista parotica (Figure A2c); quadratojugal well developed; angle between the ventral ramus of the squamosal and the quadratojugal about 40°;

maxillary exhibits a conspicuous pre-orbital process consisting of a small and pointed anterior part and a longer flap-like posterior part attending but not reaching palatinum dorsolaterally (not discernable in the cleared and stained preparations; only recognizable in X-ray computed micro tomography); maxillaries bear about 40 conical teeth with pointed tips, fore teeth remarkably longer, more robust, more pointed and more curved than the shorter and straighter rear ones (Figure A2c, A9a); premaxillaries also comprising long, robust, curved and pointed teeth, 5 on the left and 6 on the right side (Figure A2d); alary process in the middle of the premaxillary directed anterodorsally, its dorsolaterally oriented ends bifurcate, palatal shelf moderately wide and proximal and distal palatal processes strongly pronounced; anterior palatal shelf (flange) of the maxillary posterodorsally oriented, expanding strongly, ending hook-like; bony septomaxillaries (Figure A2c) with strikingly thin and pointed posteroventral processes; mentomeckelian on its border to the dentary with a conspicuous, posteriorly curved tusk-like odontoid tooth; angulosplenial with rounded coronoid process; alary processes of the parasphenoid long, narrow and posteriorly directed (Figure A2b); angle between cultriform process and alary process about 110°; cultriform process half as wide as posterior sphenethmoid, reaching up to the middle of the sphenethmoid anteriorly; a prominent posteromedial process of the parasphenoid reaching almost the foramen magnum; posterior margin of the neopalatine elevated; shape of the vomer complex (Figure A2b; A8a), its posteromedial ramus (dentigerous process) covering the proximal part of the neopalatine, five teeth present; posterior process of vomer well developed, connecting to main mass of vomer.

Vertebral column and ilia: Vertebral column (Figure A2f) consisting of seven procoelus presacral vertebrae, eights vertebra rather amphicoelous; atlas in dorsal view compact with cuspidal cervical cotyles, in ventral view centrum of atlas clearly wider than centra of all other vertebrae; relative length of the transverse processes: III>IV≈V≈VI≈VII≈VIII>II, those of vertebra II slightly anteriorly directed, those of III, IV, V and VI slightly posteriorly oriented and those of vertebra VII straight; sacral diapophyses scarcely dilated, posteriorly directed; angle between the central axis of the urostyle and the central axis of a sacral diapophyse about 58°, a roof-like protuberance from their proximal ends to the neural arch; neural arches and even the neural processes of all vertebrae do not overlap (nonimbricate state); vertebrae II-VII with well-developed neural processes, vertebrae I and VIII without such neural processes; urostyle with dorsal crest, crest proximally with an elongate knot and cleft dorsally over most of its length, urostyle possessing bicondylar articulation to the sacrum; ilium with well pronounced dorsal crest (about as high as the ilial shaft) that reaches almost to the end of the bone.

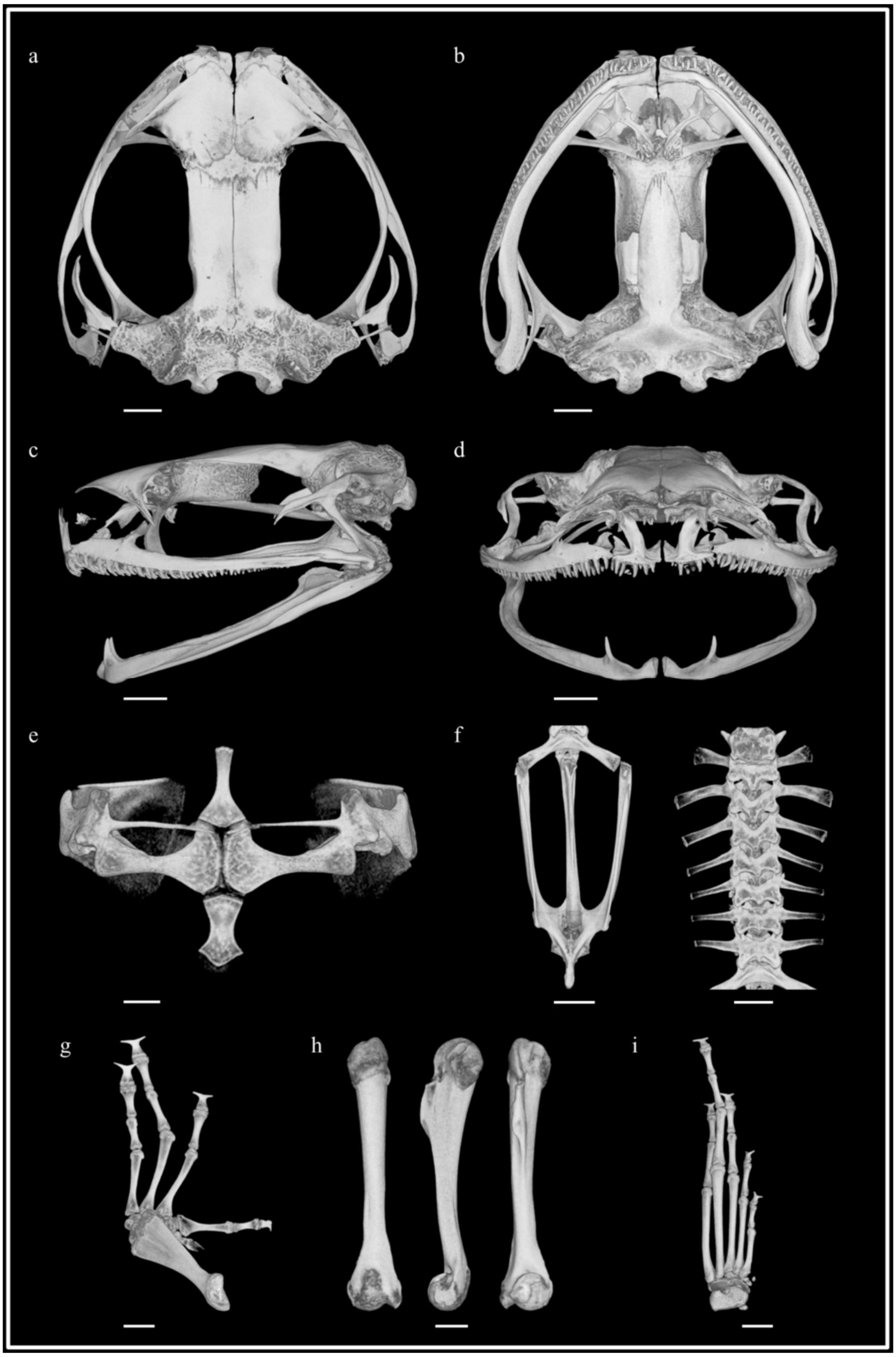


**Figure A2 Osteological details of *Odontobatrachus natator*.** Osteological details of *Odontobatrachus natator* (ZMB 78203); virtually isolated. a: dorsal view of the skull, scale bar = 2mm; b: ventral view of the skull, scale bar = 2mm; c: lateral view of left side the skull, lower jaw virtually rotated to open the mouth, scale bar = 2mm; d: frontal view of the skull, lower jaw virtually rotated to open the mouth, scale bar = 2mm; e: ventral view of pectoral girdle, scale bar = 1.5mm; f: axial skeleton in dorsal view (left = pelvic girdle, scale bar = 3mm; right = vertebral column, scale bar = 2mm); g: left hand in dorsal view, unguals virtually positioned in plane, scale bar = 2mm; h: left humerus (left = dorsal view; middle = lateral view; right = ventral view), scale bar = 1.5mm; i: left foot in dorsal view, unguals virtually positioned in plane, scale bar = 2.5mm.

Pectoral girdle: pectoral girdle firmisternal (Figure A2e); coracoids robust, perpendicular to each other and fused with the scapulae, their proximal ends not overlapping, proximal and distal epiphyses of the same size; omosternum completely ossified, base of omosternum convex and not divided as in many other African ranids [7,8]; episternum cartilaginous with shape of transversal section of a mushroom whose “head´s” centre is slightly concave and whose stipe extends anteriorly; clavicle with broad base that is united with the pars acromialis of the scapula, proximal ends of clavicles lie close to one another, not fused to the coracoids; scapula and cleithrum with adjacent areas ossified, only posterior part of the suprascapula moderately cartilaginous; procoracoids small, cartilaginous, rod-shaped elements, distally invested to the scapulae and proximally terminating in the half length of the posterior side of the clavicles (in 3 of 4 cases: right procoracoid of ZMB 78203 reaches up to the proximal tip of the clavicle); distal parts of procoracoids stronger developed than proximal parts (proximal part of the procoracoids are more strongly developed in most other anurans); bony metasternum short and wide, its lateral surfaces concave, its distal margin almost triangular; cartilaginous and bilobate xiphisternum somewhat smaller than episternum.

Extremities: humerus bears a short and rounded crista on its proximal anterior half; large cristae on male humerus absent (Figure A2h); phalangeal formula of the hand 2-2-3-3 (Figure A2g); terminal phalanx of every finger and toe T-shaped and downward oriented; no peculiarities in the shape or structure of the long bones of the hind limb; phalangeal formula of the foot 2-2-3-4-3 (Figure A2i); metacarpal of digit II not differing in shape.

Hyolaryngeal apparatus (Figure A3): hyoid plate wider than long; hyale without a free flange towards jaw, medial element of anterior process (anterior horn) of hyale small and hook-like; hyoglossal sinus deeper than anterior border of base of alary processes; anterolateral process T-shaped with a broad base, the posterolateral processes long, reaching up to the middle of the posteromedial processes; distance between posteromedial processes less than one time the width of proximal expansion of posteromedial processes apart; small cartilaginous bridge between the enlarged anterior ends of the posteromedial processes; calcifications or ossifications only in posteromedial processes present.

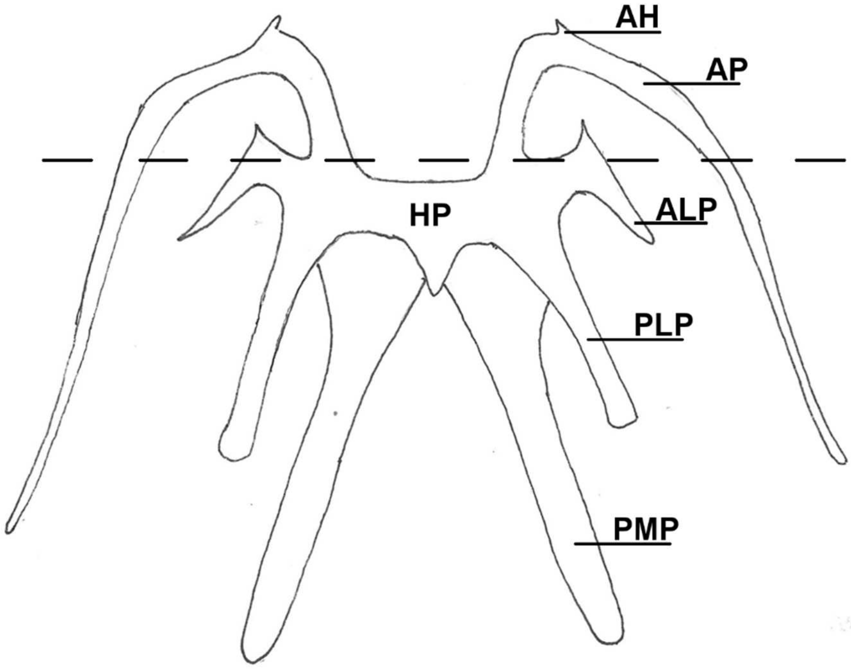


**Figure A3 Hyoid of *Odontobatrachus natator*.** Hyoid of *Odontobatrachus natator* (ZMB 78222); AH = anterior horn, ALP =anterolateral process, AP = anterior process, HP = hyoid plate, PLP = posterolateral process, PMP = posteromedial process (thyrohyal), dashed line showing to hyoglossus sinus.

### 2.2 Osteological characters distinguishing Petropedetidae and Odontobatrachidae fam. nov.

Using micro-tomographic analysis we differentiated *Odontobatrachus* from formerly assumed close relatives of the genera *Petropedetes*, and *Arthroleptides* . Applied scanning specifications as in Material and Methods section. Skeletal features of *Odontobatrachus* have been compared to following taxa: *Arthroleptides martiensseni* (ZMB 21793; male holotype), *A. yakusini* (ZMB 48472; female), *Petropedetes cameronensis* (ZMB 8222; female holotype, ZMB 27159 male syntype of *P. obscurus* = *P. cameronensis* see Barej et al. 2010), *P. euskircheni* (ZMB 73693; male paratype), *P. perreti* (ZMB 73735; male), *P. vulpiae* (ZMB 73692; male paratype); *P. juliawurstnerae* (ZMB 73694; male paratype). In her simultaneous analysis of molecular and morphological characters in ranid frogs, Scott (2005) provided osteological characters of the family Petropedetidae based on X-rays photos and cleared and stained preparations. Her material consisted of *Arthroleptides martiensseni*, *Petropedetes* (now: *Odontobatrachus*) *natator*, *Petropedetes cameronensis, P. parkeri* and *P. newtoni.* In their revision of Central African torrent frogs Barej et al. [9] synonymised *P. newtoni* with *P. johnstoni* and described three new taxa. Vouchers examined by Scott [8] were not available to us; hence, species assignments cannot be reviewed to ensure comparison with our material. Recognized

differences between Odontobatrachidae fam. nov and Petropedetidae are summarized on genus level in Table A5 and illustrated in Figures A3-A15, respectively.

**Table A5 Osteological characters in African torrent-frogs (part1).** Distinguishing osteological characters of the families Petropedetidae and Odontobatrachidae fam. nov. Characters assessed by CT-scans. Continued on next page.

| | **Odontobatrachidae fam. nov.** | **Petropedetidae** | | |
|---|---|---|---|---|
| | ***Odontobatrachus*** | ***Petropedetes*** | ***Arthroleptides*** | **see figure** |
| | | **axial skeleton and pectoral girdle** | | |
| clavicle, thickness | thin, tapering medially and meeting the procoracoid cartilage medially | thin or of moderate thickness, not tapering medially meeting the procoracoid cartilage medially | approximately equal in width along entire length, of moderate thickness, meeting the procoracoid cartilage medially | A4 |
| medial edges of coracoids | not overlapping | not overlapping | overlapping | A4 |
| posterior section of metasternum, shape | wide, triangular and weakly serrated | wide, triangular with distinctly serrated posterior edge | narrow, not serrated, hardly triangular or straight | A4 |
| ossification of suprascapular cartilage | limited to moderate (only ZMB 78243) ossification | considerable ossification | moderate to considerable ossification | A4 |
| omosternum, shape of proximal end (base) | convex | concave to bifurcated | bifurcated | A5 |
| | | **skull** | | |
| ventral sphenethmoid, extent of forward expansion of mineralized anterior portion | considerable expansion | no expansion | no expansion | A6 |
| parasphenoid alae, in ventral view | clearly angled posteriorly | perpendicular to body axis (lateral) | perpendicular to body axis (lateral) | A6 |
| pterygoid, anterior ramus in relation to neopalatines and planum antorbitale | not reaching neopalatines and planum orbitale | reaching neopalatines and planum orbitale | reaching neopalatines and planum orbitale | A7 |
| neopalatines | medially not broaded | distinctly broadend | medially not broaded | A8 |
| vomers, development and position | not reduced | reduced and seperated in two parts | reduced and solely anterior part present | A8 |
| vomer, posterior process | well developed, connected to main mass of vomer | separate from main mass of vomer | absent | A8 |
| vomerine teeth | present | present | absent | A8 |
| pre- and maxillary teeth | distinct, pointed and curved backwards, of tooth-like shape | straight or slightly backwards oriented cone-like processes | straight or slightly backwards oriented cone-like processes | A9 |
| tusk-like odontoid on mandible | present in males and females | absent | absent | A10 |

**Table A4 Osteological characters in African torrent-frogs (part 2).** Continue of Table A4

| | **Odontobatrachidae fam. nov.** | **Petropedetidae** | | |
|---|---|---|---|---|
| | ***Odontobatrachus*** | ***Petropedetes*** | ***Arthroleptides*** | **see figure** |
| | | **skull (continued)** | | |
| nasals, median contact | in median contact, with exception of one individual | widely separate, not in contact | widely separate, not in contact | A11 |
| nasals, shape | broad, triangular to rectangular, with short rear process | slender triangular, with prolonged rear process | slender triangular, with prolonged rear process | A11 |
| frontoparietals, anterior margins | medial edges extend forward nearly as much as lateral edges | medial edges do not extend forward as much as lateral edge | medial edges do not extend forward as much as lateral edge | A12 |
| premaxilla, angle of pars facialis (alary process), transverse plane | inclined laterally outwards away from midline | dorsally, perpendicular to pars dentalis | dorsally, perpendicular to pars dentalis | --- |
| squamosal, length of zygomatic ramus relative to length of otic ramus | zygomatic ramus longer than otic ramus | zygomatic ramus approximately equal in length or shorter than otic ramus | zygomatic ramus shorter than otic ramus | A13 |
| | | **extremities** | | |
| bifurcate cristae on distal dorsal surface of humerus in males | absent | present (not all species) | present (not all species) | A14 |
| metacarpal of digit II in breeding males forming a spike | absent | present (not all species) | present (not all species) | A15 |

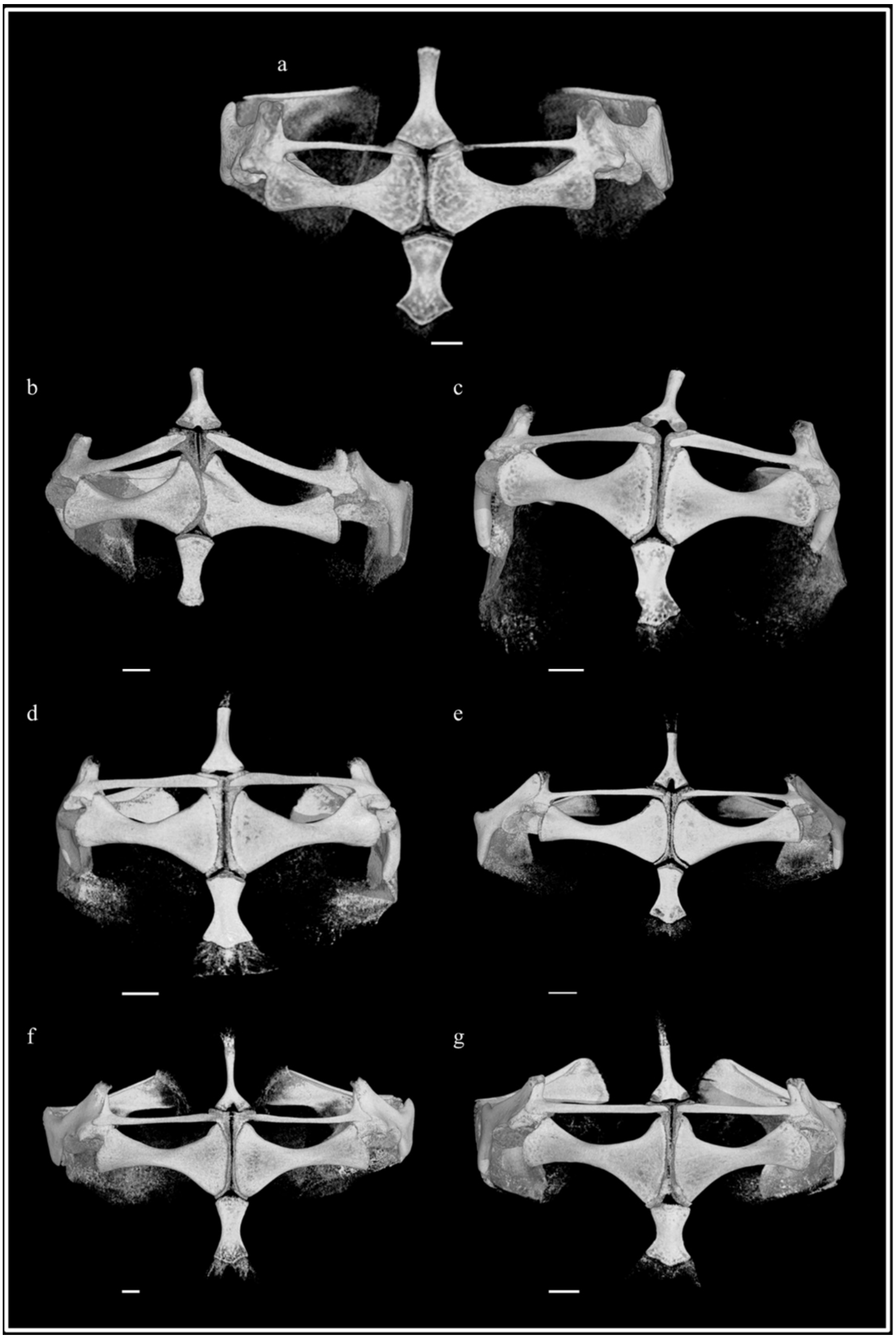


**Figure A4 Pectoral girdle in African torrent-frogs.** Ventral view of the pectoral girdle; virtually isolated; scale bar = 1mm. a: *Odontobatrachus natator* (ZMB 78203); b: *Arthroleptides martiensseni* (ZMB 21793), coracoids disarticulated; c: *Petropedetes cameronensis* (ZMB 8222), posterior section of metasternum incomplete; d: *P. perreti* (ZMB 73735); e: *P. juliawurstnerae* (ZMB 73694); f: *P. euskircheni* (ZMB 73693); g: *P. vulpiae* (ZMB 73692).

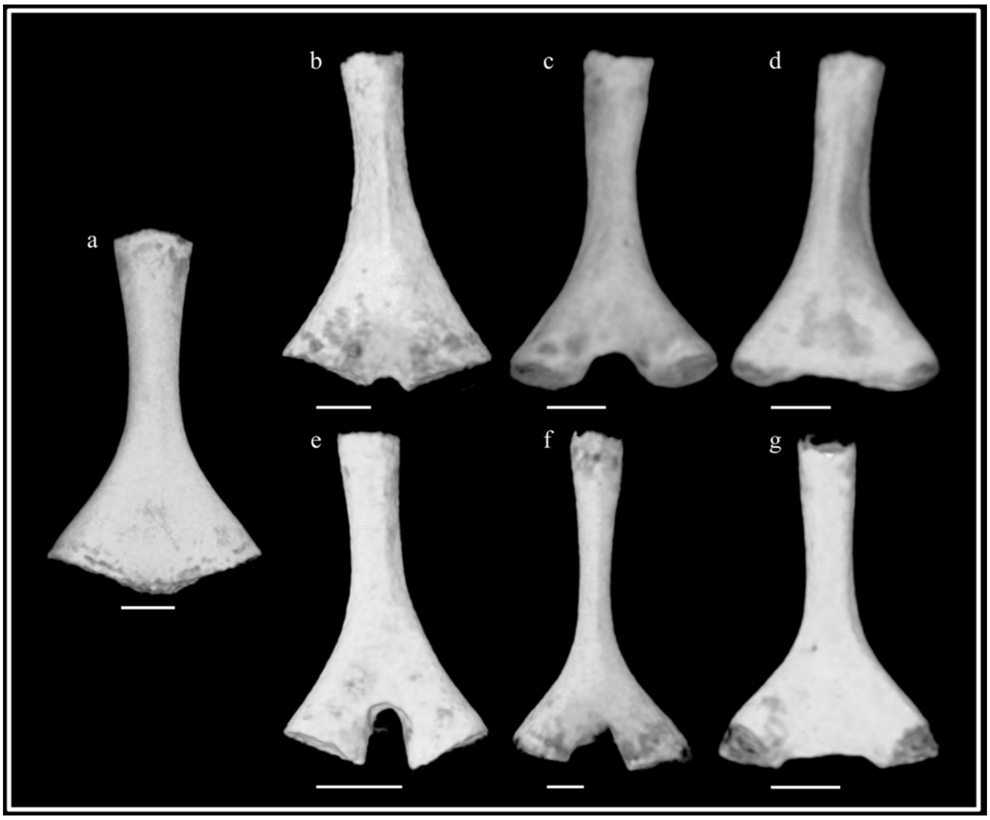


**Figure A5 Omosternum in African torrent-frogs.** Omosternum in ventral view; virtually isolated; scale bar = 0.5mm. Species assignments as in Figure A4.

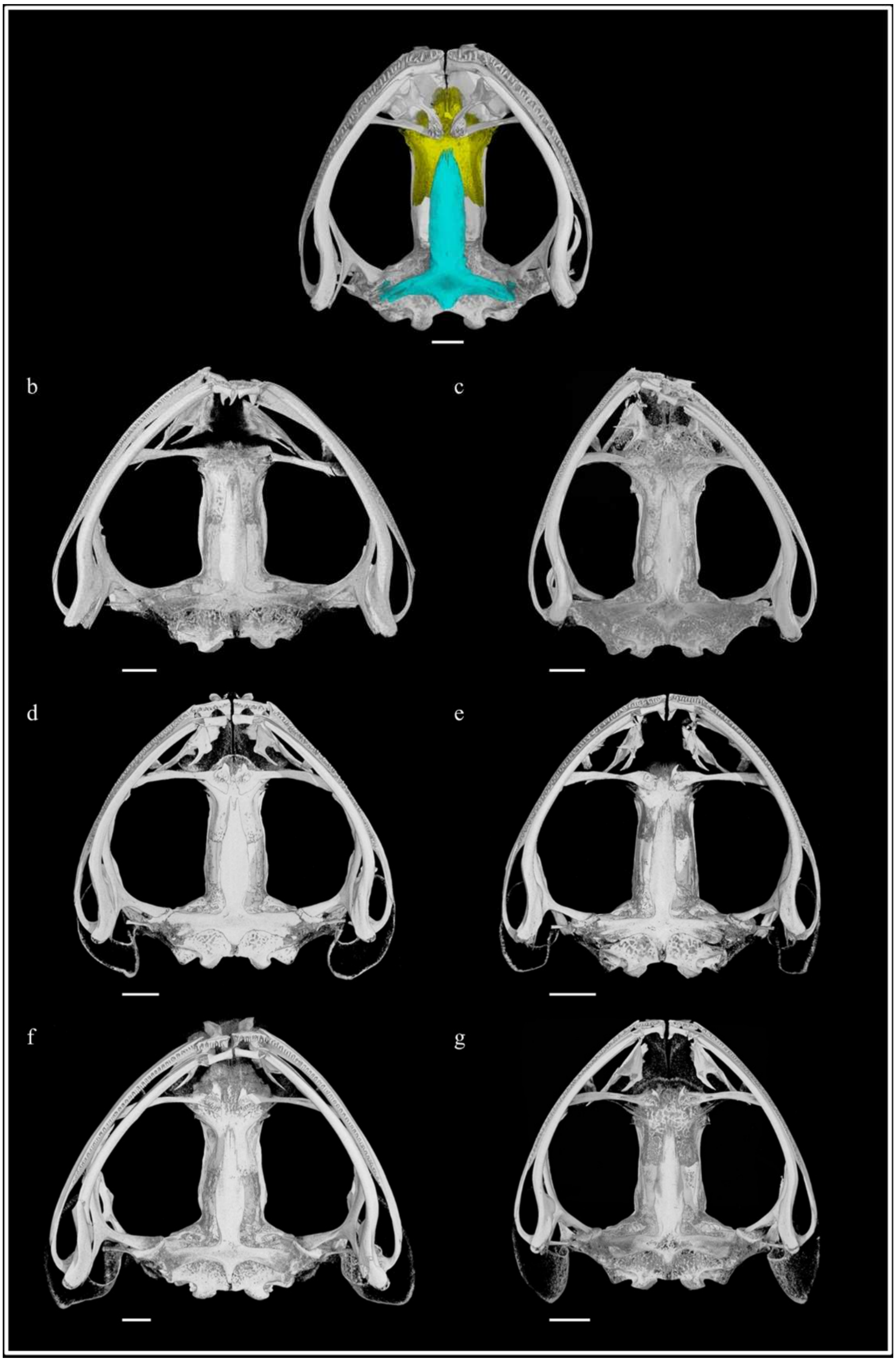


**Figure A6 Ventral view of skull in African torrent-frogs.** Ventral view of the skull; virtually isolated; scale bar = 2mm. Segmentation: yellow = sphenethmoid; blue = parasphenoid. Species assignments as in Figure A4.

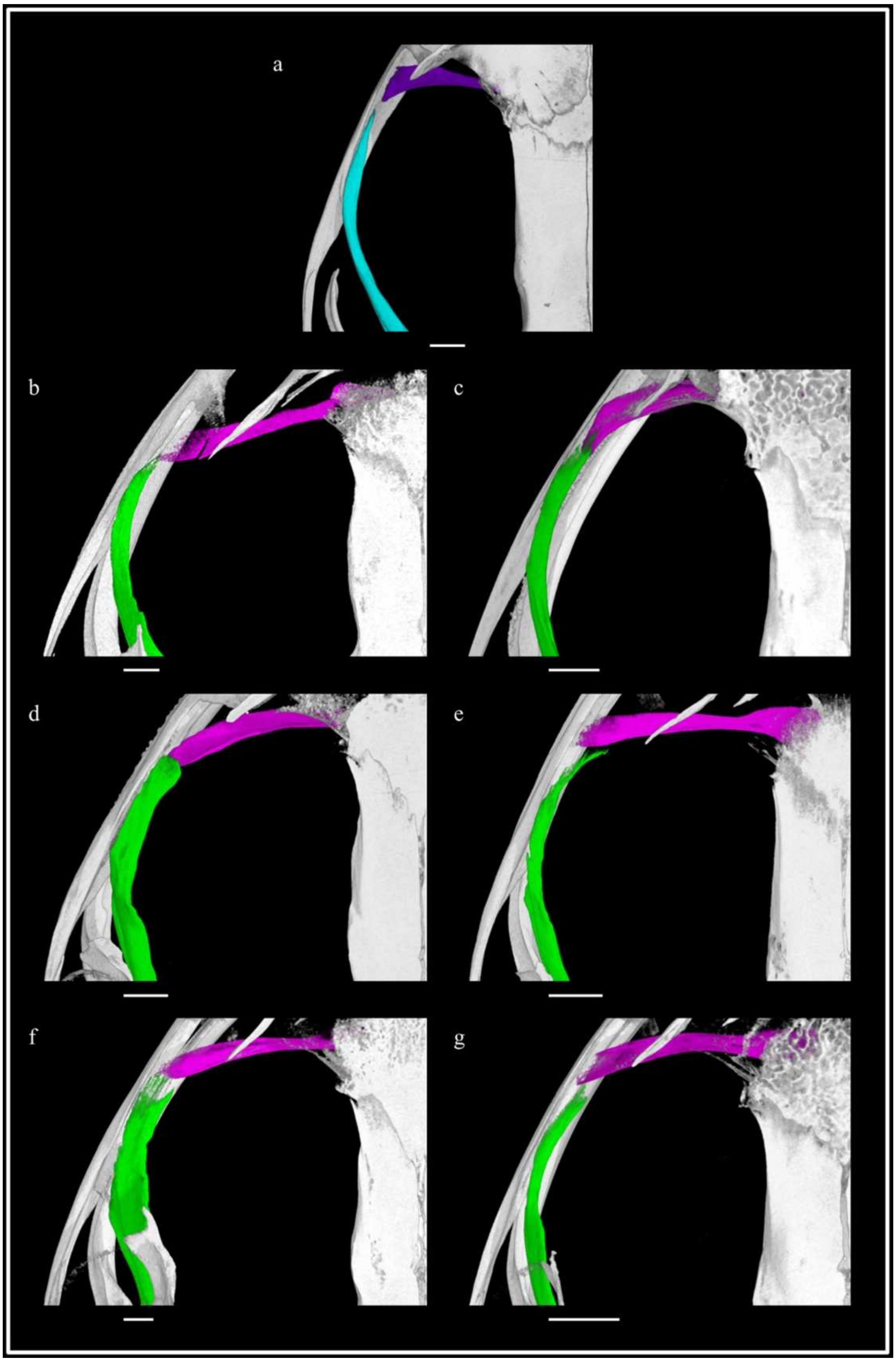


**Figure A7 Neopalatine and pterygoid in African torrent-frogs.** Close up of the left side of the skull in dorsal view; scale bar = 1mm. Segmentation: violet and red = neopalatine; blue and green = pterygoid. Species assignments as in Figure A4.

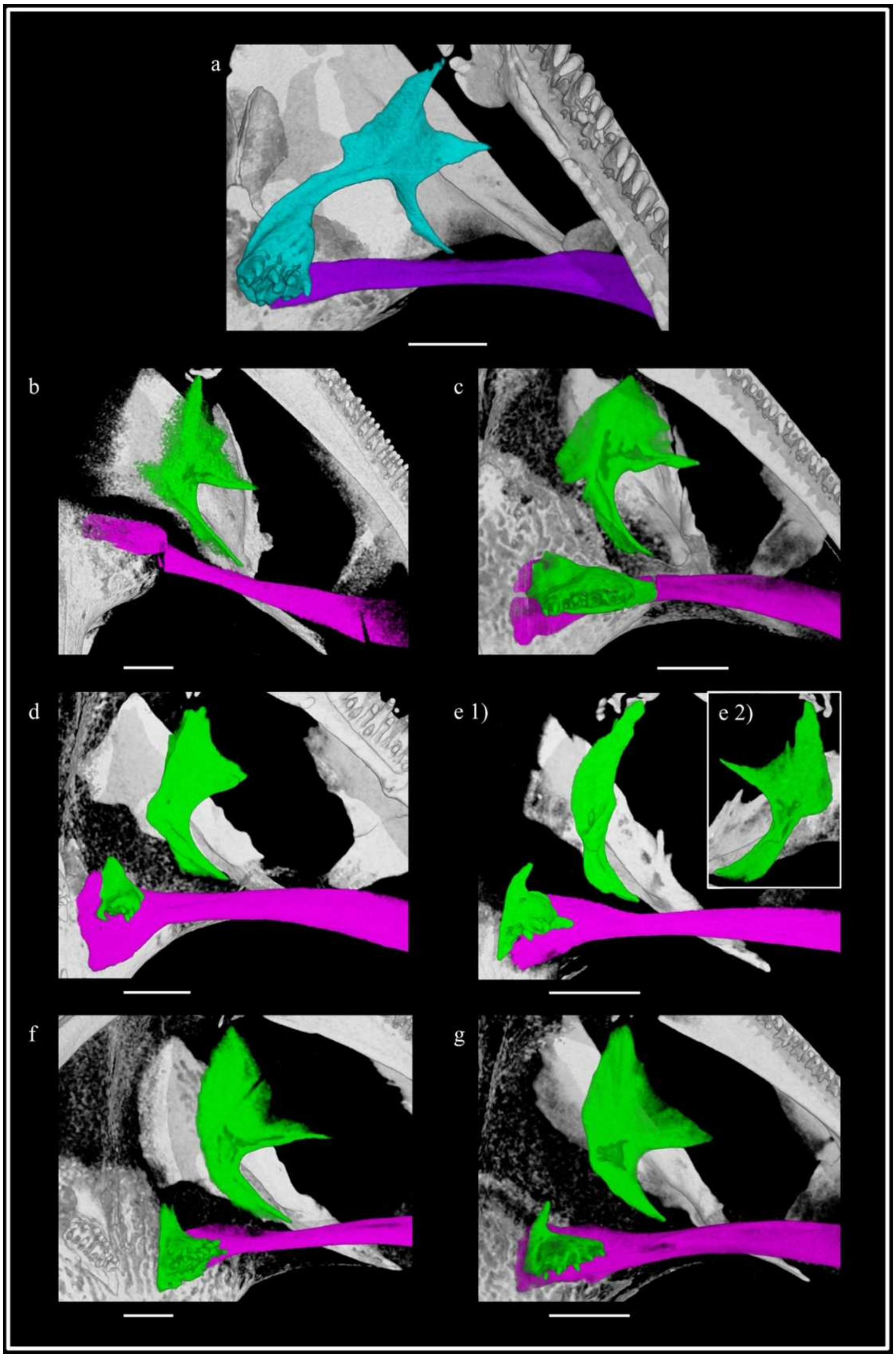


**Figure A8: Vomerine teeth in African torrent-frogs.** Close up of the left side of the skull in ventral view, exceptionally e2): right side; scale bar = 1mm. Segmentation: violet and red = neopalatine; blue and green = vomer and if available vomerine teeth. Species assignments as in Figure A4. Remark: e1): broken vomer of the left side of the skull of *Petropedetes juliawurstnerae* (ZMB 73694); e2): complete vomer of the right side of the skull of the same specimen.

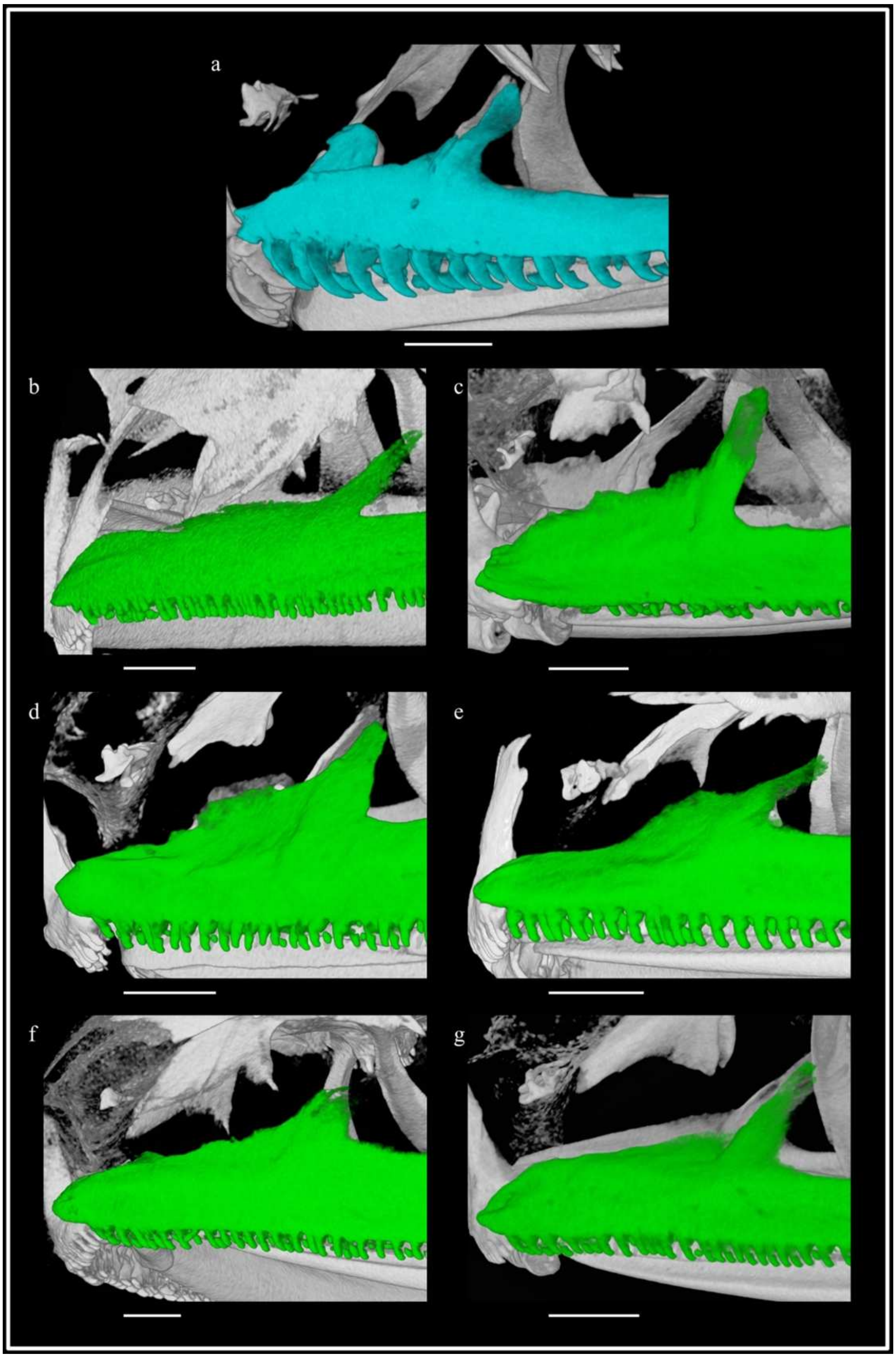


**Figure A9 Maxillary teeth in African torrent-frogs.** Close up of the anterior part of the skull in lateral view, left side of the skull; scale bar = 1mm. Segmentation: blue and green = the anterior third of the maxilla. Species assignments as in Figure A4.

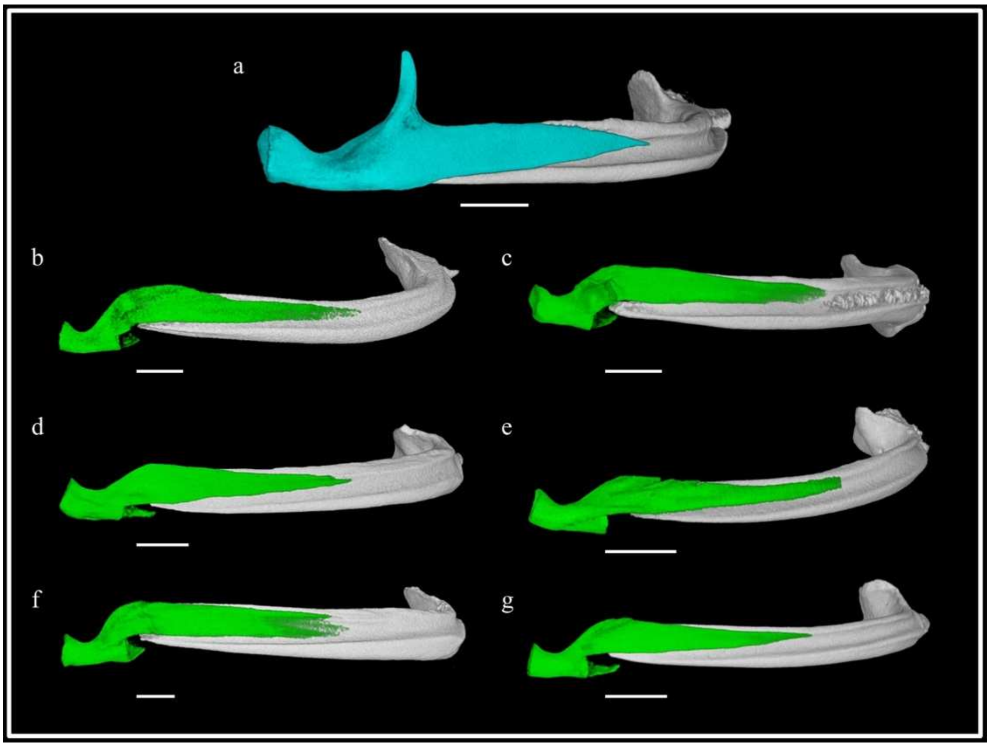


**Figure A10 Left mandible in African torrent-frogs.** Left mandible in anterior view; virtually isolated; scale bar = 1mm. Segmentation: blue and green = mentomeckelian and dentary bone. Species assignments as in Figure A4.

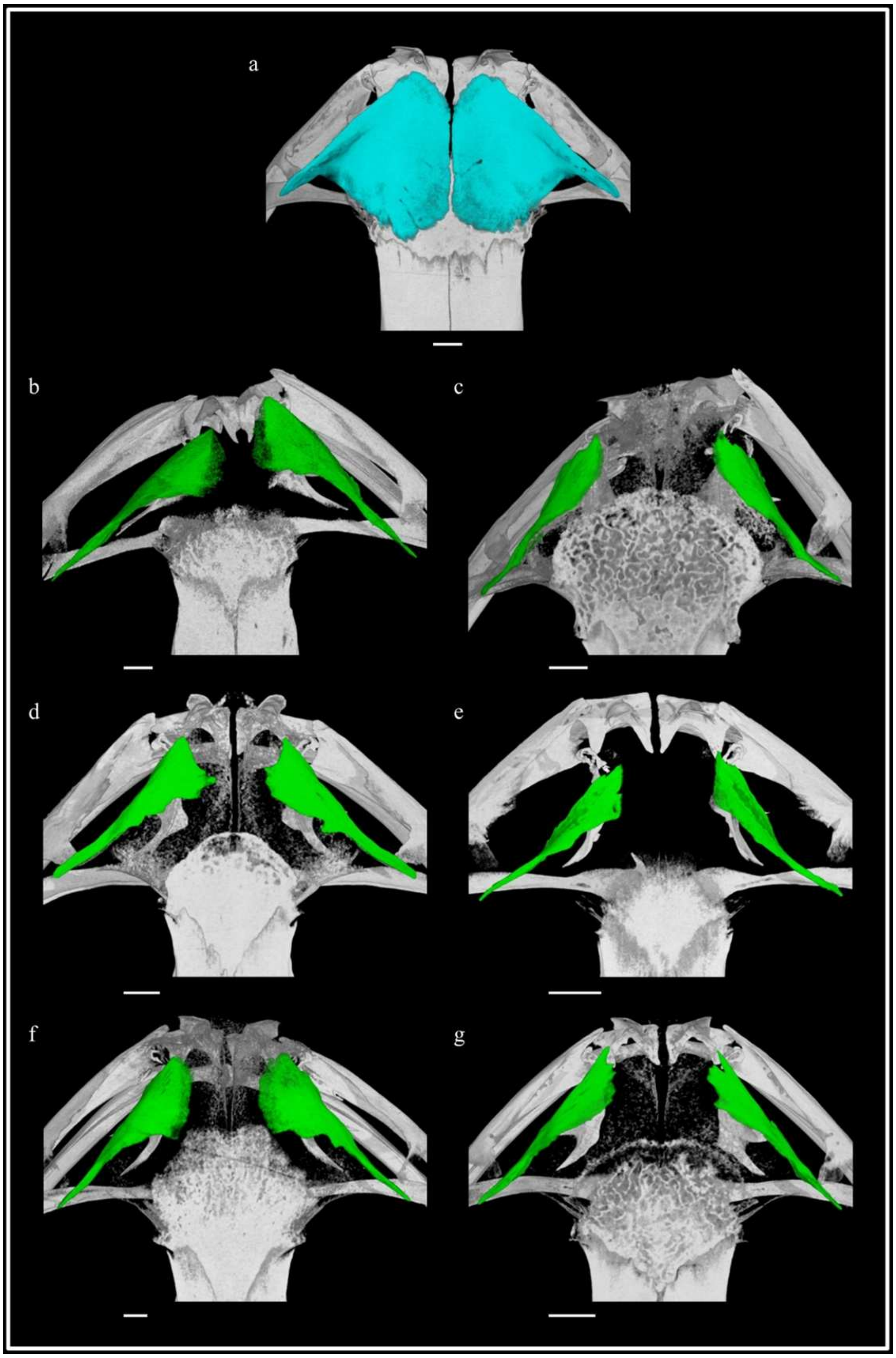


**Figure A11 Shape of nasal bones in African torrent-frogs.** Close up of the anterior part of the skull in dorsal view; scale bar = 1mm. Segmentation: blue and green = nasalia. Species assignments as in Figure A4.

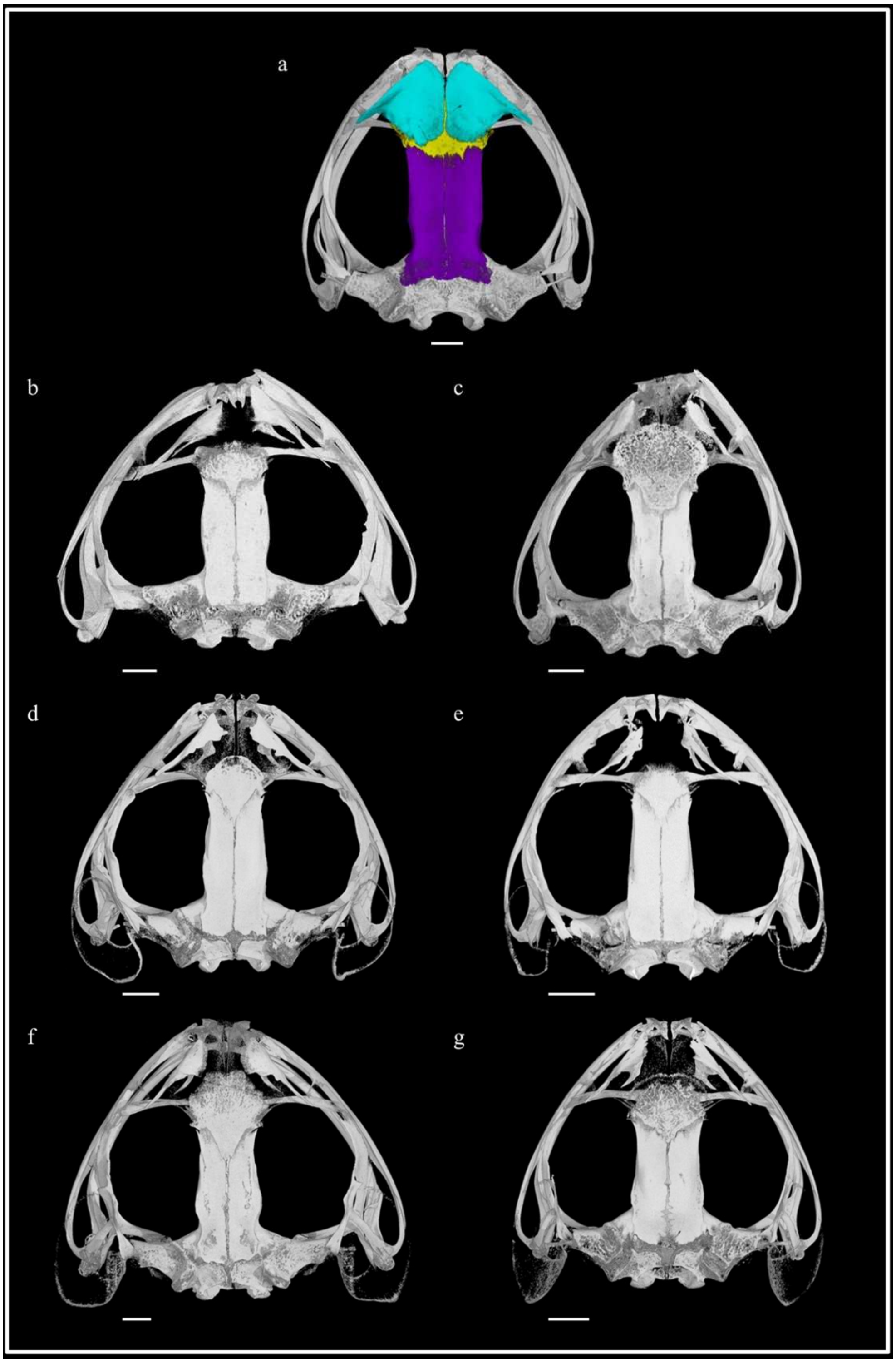


**Figure A12 Skull in dorsal view in African torrent-frogs.** Skull in dorsal view; virtually isolated; scale bar = 2mm. Segmentation: blue = nasalia; yellow = sphenethmoid; violet = frontoparietals. Species assignments as in Figure A4.

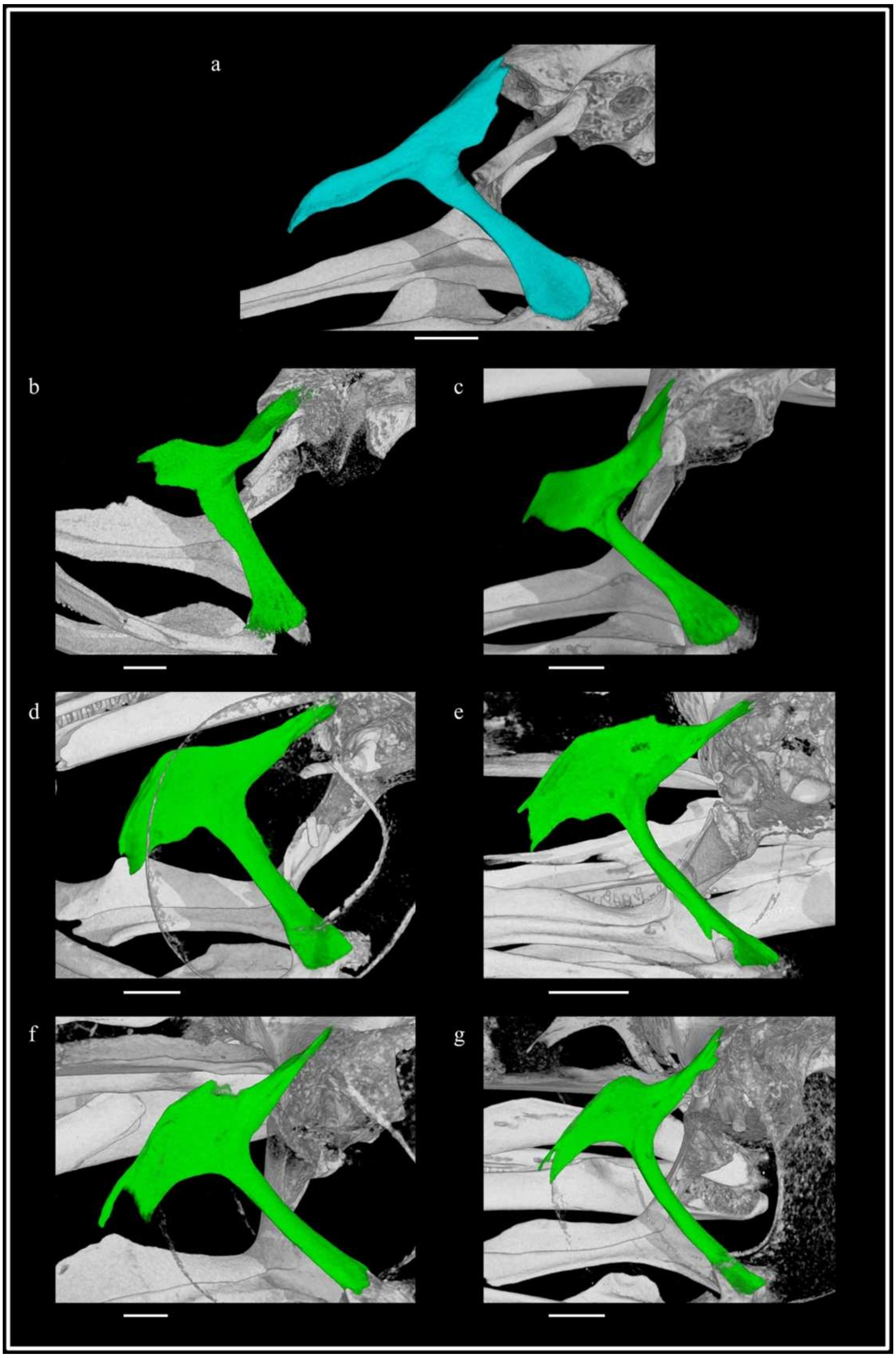


**Figure A13 Squamosal in African torrent-frogs.** Close up of the left side of the skull; anterolateral view, until the squamosal reaches the maximum expansion; scale bar = 1mm. Segmentation: blue and green: squamosal. Species assignments as in Figure A4.

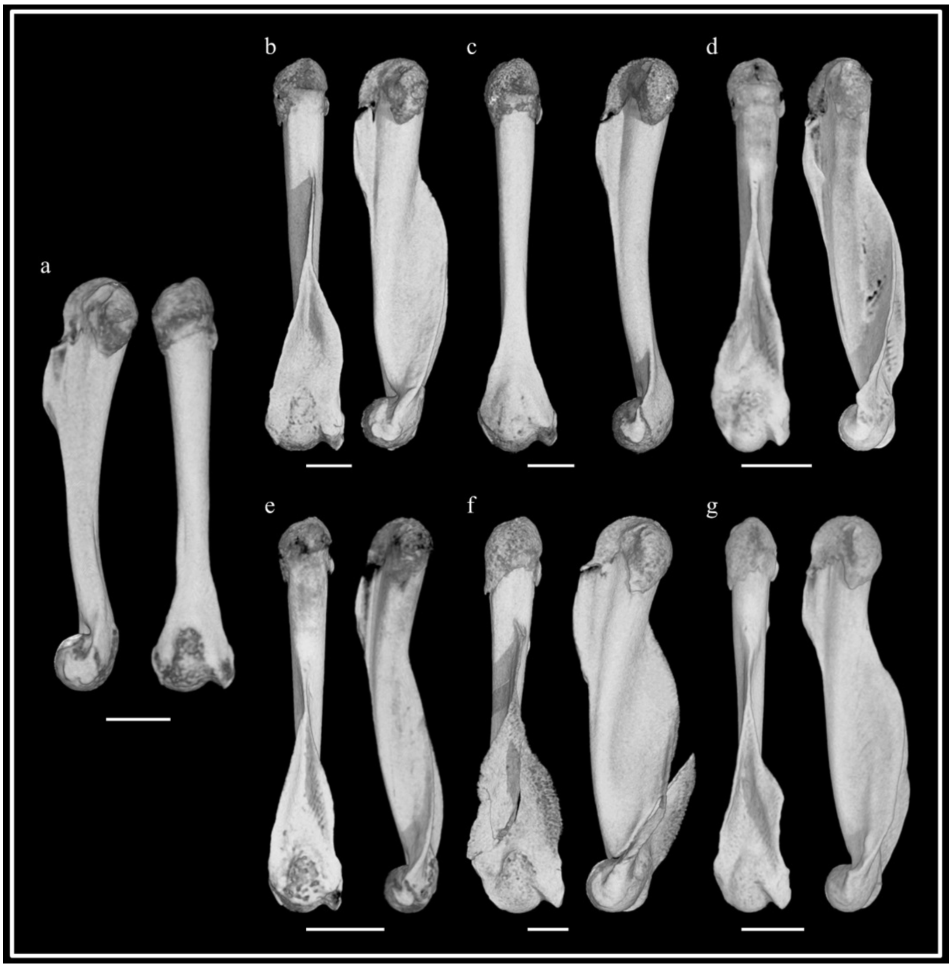


**Figure A14 Humerus in African torrent-frogs.** Humerus virtually isolated, left = dorsal view, right = lateral view, positioned anterior to posterior from top to bottom, scale bar = 2mm. Species assignments as in Figure A4; *Petropedetes cameronensis* (ZMB 27159). *P. cameronensis* lacks the enlarged crista as brachial hypertrophy is not present in the species [9]; comparable to the on-related genus *Leptodactylodon* [10], in which males of species with brachial hypertrophy likewise possess enlarged cristae on the humerus.

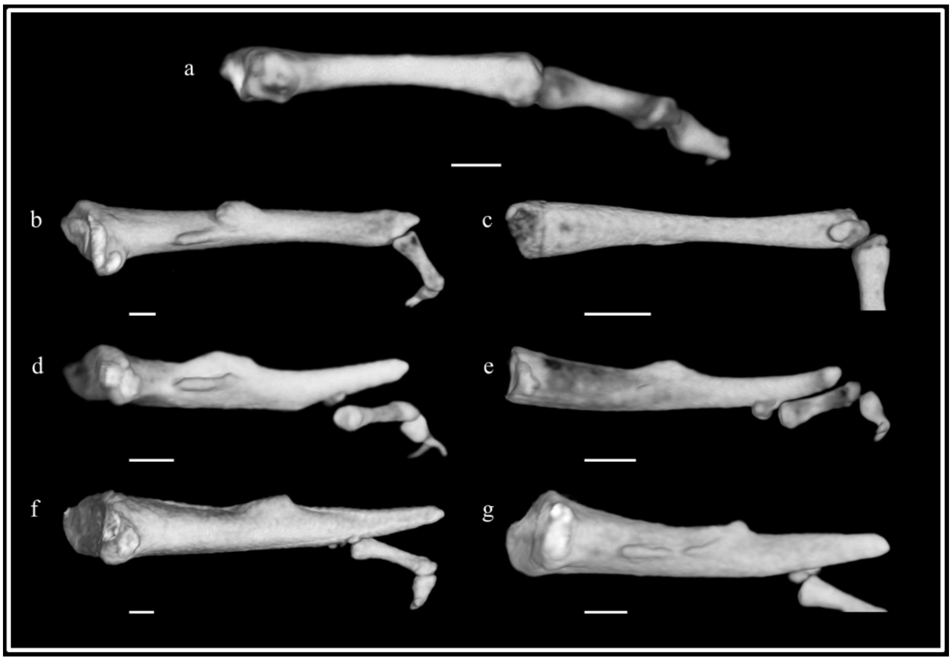


**Figure A15 Metacarpal of Digit II in African torrent-frogs.** Close up of the left hand, metacarpal of Digit II in lateral view; virtually isolated, scale bar = 0.5 mm. Species assignments as in Figure A4; *Petropedetes cameronensis* (ZMB 27159). *P. cameronensis* lacks a spike [9].

## 3. Amphibian diversity in African realms

In order to get an overview of overall diversity and number of endemic taxa, numbers of families and genera occurring in the Upper Guinea forests, we compared endemicity on different systematic levels in the four African biogeographic regions of western, central, eastern and southern Africa as recognized in published literature [11-16]. We refer to rough geographic sub-divisions, as present knowledge is incomplete, differing between vertebrate groups and applied data [16,17]. Sub-division is as follow: western Africa: Senegal to west of Cross River, central Africa: east of Cross River, eastwards to the Albertine Rift, southwards to Angola and north-western Zambia, eastern Africa: east of the Albertine Rift, southwards to Mozambique, southern Africa: south of central and eastern Africa, northwards to Ethiopia and Eritrea. Borders of recognized areas of central, eastern and southern Africa have not been demarcated strictly in the literature however such border areas do not contain endemics above the species level. Table A6 provides data on shared higher taxa between at least two biogeographic regions, endemics and total numbers at generic and family level are given (Table A6). Data extracted from Frost [18].

**Table A6 Amphibian diversity in African biogeographic regions.** Amphibian diversity (on family and genus level) in African realms based on Frost [18].

| _ | western Africa | Central Africa | eastern Africa | southern Africa |
|---|---|---|---|---|
| genera (shared) | 24 | 28 | 27 | 24 |
| genera (endemic) | 3 | 17 | 13 | 11 |
| genera (total) | 27 | 45 | 40 | 35 |
| | | | | |
| families (shared) | 13 | 14 | 15 | 13 |
| families (endemic) | 1 | 0 | 0 | 1 |
| families (total) | 14 | 14 | 15 | 14 |

## 4. References


1. Kotaki M, Kurabayashi A, Matsui M, Khonsue W, Hon Djong T, Tandon M, Sumida M: **Genetic divergences and phylogenetic relationships among the *Fejervarya limnocharis* complex in Thailand and neighboring countries revealed by mitochondrial and nuclear genes.** *Zool Sci* 2008, **25:**381-390.
2. Kotaki M, Kurabayashi A, Matsui M, Kuramoto M, Hon Djong T, Sumida M: 2010. **Molecular phylogeny of the diversified frogs of genus *Fejervarya* (Anura: Dicroglossidae).** *Zool Sci* 2010, **27:**386-395.
3. Benson DA, Cavanaugh M, Clark K, Karsch-Mizrachi I, Lipman DJ, Ostell J, Sayers EW: **GenBank.** *Nucleic Acids* 2013, **41:** D36-D42.
4. Bossuyt F, Roelants K: **Frogs and toads (Anura).** In *The time-tree of life.* Edited by Hedges SB, Kumar S Oxford University Press; 2009: 357-364.
5. Avise JC, Liu J-X: **On the temporal inconsistencies of Linnean taxonomic ranks.** *Biol J Linn Soc* 2011, **102:**707-714.
6. Trueb L: **Bones, frogs, and evolution.** In: *Evolutionary biology of the anurans: contemporary research on major problems*. Edited by Vial JL. University of Missouri Press, Columbia, Missouri, 1973:65-132.
7. Deckert K: **Beiträge zur Osteologie und Systematik ranider Froschlurche.** *Sber Ges naturf Freunde Berl* 1938, **4–7:**127-184.
8. Scott E: **A phylogeny of ranid frogs (Anura: Ranoidea: Ranidae), based on a simultaneous analysis of morphological and molecular data.** *Cladistics* 2005, **21:**507-574.
9. Barej MF, Rödel M-O, Gonwouo NL, Pauwels OSG, Böhme W, Schmitz A: **Review of the genus *Petropedetes* Reichenow, 1874 in Central Africa with the description of three new species (Amphibia: Anura: Petropedetidae).** *Zootaxa* 2010, **2340:**1-49.
10. Amiet J-L: **Révision du genre *Leptodactylodon* Andersson (Amphibia, Anura, Astylosterninae).** *Ann Fac Sci Yde* 1980, **27:**69-224.
11. Channing A: *Amphibians of Central and Southern Africa*. Ithaka: Cornell University Press; 2001.
12. Channing A, Howell KM: *Amphibians of East Africa*. Ithaka: Cornell University Press; 2006.
13. du Preez L, Carruthers V: *A complete guide to the frogs of southern Africa.* Cape Town: Striuk nature; 2009.

14. Largen M, Spawls S: *The amphibians and reptiles of Ethiopia and Eritrea*. Frankfurt/M: Chimaira Edition; 2010.
15. Penner J, Wegmann M, Hillers A, Schmidt M, Rödel M-O: **A hotspot revisited – a biogeographical analysis of West African amphibians.** *Divers Distrib* 2011, **17:**1077-1088.
16. Poynton JC: **Distribution of amphibians in Sub-Saharan Africa, Madagascar, and Seychelles.** *In Patterns of distribution of amphibians, a global perspective.* Edited by Duellman WE: Johns Hopkins University Press, Baltimore; 1999:483-539.
17. Linder HP, de Klerk HM, Born J, Burgess ND, Fjeldså J, Rahbek C: **The partitioning of Africa: statistically defined biogeographical regions in sub-Saharan Africa.** *J Biogeograph* 2012, **39:**1189-1205
18. Frost DR: Amphibian Species of the World: an Online Reference. Version 5.6. [http://research.amnh.org/herpetology/amphibia/index.html] 2013.
